# Serotonin (5-HT) 2A Receptor Involvement in Melanin Synthesis and Transfer via Activating the PKA/CREB Signaling Pathway

**DOI:** 10.3390/ijms23116111

**Published:** 2022-05-30

**Authors:** Yunyun Yue, Min Zhong, Xiaohong An, Qingyuan Feng, Yifan Lai, Meng Yu, Xiaofeng Zhang, Zixian Liao, Minghan Chen, Jing Dong, Hui Zhong, Jing Shang

**Affiliations:** 1 School of Traditional Chinese Pharmacy, China Pharmaceutical University, Nanjing 210009, China; 1620184488@cpu.edu.cn (Y.Y.); zhongmin18111@163.com (M.Z.); 15298366363@163.com (X.A.); 15651715868@163.com (Q.F.); laiyifan1027@163.com (Y.L.); yumeng0701@163.com (M.Y.); 15521076223@163.com (X.Z.); liaozixianpharm@163.com (Z.L.); 13859874612@163.com (M.C.); dongjingtx@gmail.com (J.D.); 2 State Key Laboratory of Natural Medicines, China Pharmaceutical University, Nanjing 210009, China; 3 Jiangsu Key Laboratory of TCM Evaluation and Translational Research, China Pharmaceutical University, Nanjing 210009, China; 4 NMPA Key Laboratory for Research and Evaluation of Cosmetics, National Institutes for Food and Drug Control, Beijing 100050, China

**Keywords:** HTR2A, melanocyte, melanogenesis, PKA/CREB signaling, zebrafish

## Abstract

The 5-HT2A serotonin receptor (HTR2A) has been reported to be involved in the serotonin- or serotonin receptor 2A agonist-induced melanogenesis in human melanoma cells. However, the molecular mechanisms underlying HTR2A in regulating melanogenesis remain poorly understood. In this research, cultured mouse melanoma cell line B16F10, human skin, and zebrafish embryos were used to elucidate the downstream signaling of HTR2A in regulating melanogenesis and to verify the potential application of HTR2A in the treatment of pigment-associated cutaneous diseases. We demonstrated that HTR2A antagonists (AT1015 and ketanserin) attenuated the melanogenesis induction of serotonin in both mouse melanoma cells and zebrafish embryos. The agonists of HTR2A (DOI and TCB-2) increased melanin synthesis and transfer in B16F10 cells, human skin tissue, and zebrafish embryos. Furthermore, the HTR2A agonists increased the expression of proteins related to melanosome organization and melanocyte dendrites to facilitate the melanocyte migration and melanosome transport. HTR2A antagonists and genetic knockout of zebrafish *htr2aa* (the homologue of mammalian *HTR2A* gene) were also used to clarify that HTR2A mediates serotonin and DOI in regulating melanogenesis. Finally, through small scale screening of the candidate downstream pathway, we demonstrated that HTR2A mediates the melanogenesis induction of its ligands by activating the PKA/CREB signaling pathway. In this research, we further confirmed that HTR2A is a crucial protein to mediate melanocyte function. Meanwhile, this research supports that HTR2A could be designed as a drug target for the development of chemicals to treat cutaneous diseases with melanocytes or melanogenesis abnormality.

## 1. Introduction

Melanogenesis is a complex biological process involving melanocyte formation, melanosome assembly and maturation, as well as melanin synthesis and transport to adjacent keratinocytes [1]. In addition to coloring our skin, the more important role of melanin in human skin is to protect DNA from UV damage [2,3]. Any abnormality during the melanin production process may lead to pigment-related cutaneous diseases, such as albinism and vitiligo with insufficient or lack of melanin, or melasma and melanoma with hyperpigmentation [4]. Therefore, identifying the crucial proteins that regulate melanogenesis to elucidate the signal regulation network of melanin production would be helpful for the development of drugs for treating dermal diseases with abnormal melanin metabolism. Current studies have shown that many nervous, immune, and endocrine factors are directly or indirectly involved in the regulation of skin melanogenesis [5]. Neurotransmitters, such as dopamine, acetylcholine, norepinephrine (NE) or epinephrine (EP), gamma-aminobutyric acid (GABA), glutamate, histamine (HA), and serotonin (5-hydroxytryptamine, 5-HT) also play key roles in melanocyte development, regeneration, and function maintenance [6,7,8].

5-Hydroxytryptamine is widely distributed in the central nervous system and is involved in regulating emotions, appetite, sleep, and multiple physiological processes. Peripheral 5-HT is mainly derived from enterochromaffin cells and stored in platelets, which circulate throughout the whole body. Recently, it has been shown that skin epidermal cells express 5-HT synthetase (TPH1) and a variety of 5-HT receptors (HTR1A, HTR1B, HTR2A, HTR5A, and HTR7) [9,10]. Exposure to sunlight or ultraviolet light increases the 5-HT level in the skin. These discoveries suggest that 5-HT plays biological roles in the physiological and pathological states of skin [11].

In recent years, it has been found that patients with congenital pigment abnormality show abnormal tryptophan metabolism and reduced blood 5-HT levels. Homozygous patients with Hermansky-Pudlak syndrome are associated with ocular and skin albinism, with platelet 5-HT levels of 15% to 20% as compared with those of the healthy population [12]. Blood 5-HT levels have also been reported to be significantly lower in patients with vitiligo as compared with healthy people. In vitro studies have shown that high doses of 5-HT (100 μM) can increase the melanin content and dendrites of B16F10, SK-MEL-2, and Melan-A cells. Our previous studies have also shown that 5-HT can induce melanogenic lineage specification from the neural crest cell during early embryonic development of zebrafish.

However, given the non-selectivity activation of serotonin receptors, it is not clear which 5-HT receptor subtype participates in the regulation of 5-HT in melanocyte development or melanin production. Mammalian 5-HT receptors are divided into 7 subfamilies containing 21 receptor subtypes, most of which are G protein-coupled receptors (GPCRs). Therefore, the spatio-temporal specific expression of these receptors is the molecular basis for the different physiological functions of 5-HT in diverse tissues or cell types. Our previous studies have shown that multiple 5-HT receptors are expressed in mouse skin, including HTR1A, HTR1B, HTR1D, HTR2A, HTR5A, HTR5B, and HTR7. In a mouse model with skin depigmentation induced by chronic mental stress, 5-HT levels in serum and skin were decreased which was similar to the situation in vitiligo patients. Moreover, the expression levels of several 5-HT receptors in the skin of mice were decreased (including HTR1A, HTR1B, HTR1D, HTR2A, HTR5A, and HTR5B) [13,14]. These studies have reported a close connection between 5-HT level and melanocyte function in vivo. However, revealing the receptor subtype that mediates 5-HT regulation of melanocyte function and elucidating its downstream molecular mechanisms are important for the treatment of skin pigmentation disorders by targeting the 5-HT signaling pathway.

Previous studies have shown that fluoxetine (reuptake inhibitors of serotonin) could regulate melanin production in B16F10 cells, zebrafish, and mice with chronic mental stress through HTR1A and HTR2A [15,16,17,18]. About ten years ago, Lee et al. found that HTR2A agonists could promote melanin production in human melanoma cell line in vitro [19]. In terms of the selectivity of small-molecular ligands and the specificity of siRNA knockdown, there is a need to further confirm the role of HTR2A in regulating melanocyte function and to elucidate the downstream molecular mechanisms. Therefore, in this study, the B16F10 cell line and zebrafish embryos were used as the main research objects to elucidate the molecular mechanisms of HTR2A mediating 5-HT and its agonists to regulate melanin synthesis and melanin transport. The results provide an important basis for us to understand the signaling regulatory network of melanin production and to develop drugs for the treatment of pigment abnormalities by targeting 5-HT receptors.

## 2. Experimental Procedures

### 2.1. Ethical Statements

The animal experiments were performed in accordance with the Jiangsu Provincial standard ethical guidelines for the use of experimental animals and were permitted by the Science and Technology Department of Jiangsu Province. All procedures performed in this study involving human participants were in accordance with the ethical standards of the institutional and/or national research committee and with the 1964 Declaration of Helsinki and its later amendments or comparable ethical standards.

### 2.2. Zebrafish Lines and Maintenance

The wild-type adult zebrafish (6–9 months old) were obtained from China Zebrafish Resource Center (Wuhan, China) and maintained in a recirculating aquaculture system at the China Pharmaceutical University. The zebrafish were maintained at 28.5 °C, under a circadian cycle of 14:10 h (light/dark). The circulating water in the aquarium was filtered by reverse osmosis (pH 7.0–7.5). The zebrafish were fed twice a day with lab-grown brine shrimp. The *htr2aa* knockout zebrafish lines *htr2aa^cpu5^* and *htr2aa^cpu6^* were constructed at the China Pharmaceutical University. The numbers of zebrafish embryos with representative phenotype in all tested embryos are showed in each figure.

### 2.3. Cell Culture and Cell Viability Assay

The B16F10 mouse melanoma cell line was purchased from the Cell Bank of the Chinese Academy of Sciences, Shanghai, China. The cells were cultured in Dulbecco’s modified Eagle’s medium (DMEM) (Gibco, Carlsbad, CA, USA) supplemented with 10% fetal bovine serum (FBS) (Gibco, Carlsbad, CA, USA) at a humidified incubator with 5% CO_2_ and 37 °C.

For the cell viability assay, the B16F10 cells were digested by trypsin and seeded into 96-well plates with about 2500 cells per well. After 24 h, the medium was changed to DMEM containing 2.5% FBS with different concentrations of the tested drugs. After incubation, MTT (5 mg/mL) was added to each well, and incubated at 37 °C for 4 h. The supernatant was discarded, 150 μL DMSO was added to each well, followed by shaking on the shaking table for 10 min. The OD value of each well was measured at 570 nm.

### 2.4. Drug Treatment and Docking Analysis

5-Hydroxytryptamine (5-HT), α-MSH, AT1015, ketanserin, PTU (N-phenylthiourea), DOI (DOI hydrochloride), TCB-2, H89, KN93, and PD909835 were used in this research. The stock concentrations and suppliers are listed in the Supplementary Materials, Table S1. The docking analysis of HTR2A protein and the ligands (5-HT, DOI, TCB-2, AT1015, and ketanserin) were performed with the MOE 2019.0102 software. The structure of five small molecules were downloaded from the PubChem Compound database based on the CAS number and name. The crystal structure of HTR2A (PDB and 6wh4) was used on the basis of the published paper [20].

### 2.5. Masson–Fontana Staining

The melanoma cells (B16F10) or skin tissue sections were fixed in 10% formaldehyde solution for 30 min, and then rinsed three times with ddH_2_O. Then, the fixed samples were stained with Fontana ammoniacal silver solution (Reagent A) (Nanjing SenBeiJia Biological Technology Co., Ltd., Nanjing, China) and soaked in the dark for 12–24 h. Then, the samples were washed with ddH_2_O three times, and subsequently, were stained with Reagent B solution for 3 min, and rinsed with ddH_2_O. The samples were counterstained with neutral red (Reagent C) for 20 min. Finally, the samples were rinsed with ddH_2_O and dehydrated in 100% ethanol, and mounted for observation under a microscope.

### 2.6. Melanin Content and Tyrosinase Activity Assay

For the measurement of tyrosinase activity, about 30 zebrafish larvae or B16F10 cells cultured in 10 cm dishes were sonicated in lysis buffer at 4 °C. The samples were centrifuged at 12,000× *g* rpm for 10 min. The supernatant was added to each well in a 96-well plate before being mixed with 190 µL 0.1% L-DOPA in 0.1 M PBS (pH 6.8) at 37 °C for 0.5 h. The optical density was measured at 475 nm. For the measurement of melanin content, the precipitate was digested in 100 µL 1 M NaOH at 80 °C for 2 h. Then, solubilized melanin was measured at 405 nm.

### 2.7. Western Blot

The B16F10 cells were washed with PBS three times, and then dissolved in 100 µL lysis buffer containing 5 µL PMSF (protease inhibitor) for 20 min. The cell lysates were centrifuged at 12,000 rpm for 15 min. Protein was collected and mixed with loading buffer and boiled at 95 °C for 10 min. Then, the 40 µg protein samples were loaded into a 10% SDS PAGE before being transferred onto PVDF membrane. Then, the membranes were blocked with 5% milk for 1 h at room temperature and incubated with the corresponding primary antibodies overnight at 4 °C. The membranes were washed three times with 1 × TBST (0.1% Tween 20), and then incubated with secondary antibody for 1 h, and visualized using enhanced chemiluminescence detection system. The relative content of the target protein was calculated using the relative gray value as compared with β-actin. The primary antibodies used for Western blot are listed in the Supplementary Materials, Table S2. The quantitation of Western blots is shown at the top of each blot.

### 2.8. Melanocyte Dendrites’ Number and Length

The B16F10 cells were cultured following a standard method. After drug treatment, the morphological photos of cells were captured by microscope. Then, the dendrites’ number and length were analyzed with the Image J software.

### 2.9. Skin Tissue Culture and H&E Staining

The foreskins of normal persons undergoing circumcision were collected in the department of urology, at the Nanjing Children’s Hospital. The skin tissue was rinsed repeatedly in DMEM medium with 1% penicillin-streptomycin and 10% FBS. The processed skin tissue was cut into 5 mm × 5 mm sections and transferred to a 24-well plate and cultured in an incubator with 5% CO_2_ and 37 °C. The cultured human skin tissue was fixed with paraformaldehyde and embedded with paraffin for sections. The skin tissue sections were stained with hematoxylin and eosin as protocol [15].

### 2.10. Generation of Zebrafish Mutants with CRISPR/Cas9

To generate zebrafish *htr2aa* mutants, we took advantage of the genome editing method by CRISPR/Cas9. The guide RNA (gRNA) was designed to target the zebrafish *htr2aa* (Gene ID 560808) coding sequence, and synthesized by transcription in vitro. Co-injection of these sgRNA (10 pg/μL) with the Cas9-NLS protein (100 pg/μL, Z03389-50, Genscript, China) resulted in small indel mutations. The sgRNA target sequences are listed in the Supplementary Materials, Table S3. After sequencing, heterozygous mutants were identified.

### 2.11. Measurement of the Pigmenting Activity in the Zebrafish

The embryos were collected in 6-well plates with 30 embryos per well, and cultured in 5 mL embryo medium. In the experiments, 0.3 mM PTU was administered from 9 to 35 hpf. After PTU washing, the embryos were added immediately with the medium with indicated concentrations of 5-HT (DOI or TCB-2) from 36–60 hpf. The effects on the pigmentation of the zebrafish were observed at 60 hpf.

### 2.12. Cytoskeleton Staining

The B16F10 cells grew on slices placed in 6-well plates. After washing 2 times with 37  °C preheated PBS, the cells were fixed for 10 min with 4% paraformaldehyde. For F-actin staining, the B16F10 cells were incubated away from light with phalloidin staining solution labelled with fluorescein isothiocyanate (Sigma, Saint Louis, MO, USA) for 30 min. The F-actin was observed using a confocal laser scanning microscope (Nikon, Tokyo, Japan). At least three fields of view were randomly selected.

### 2.13. Cell Scratch Assay

The B16F10 cells were cultured in 6-well plates, with a density of 5 × 10^5^ cells per well. When the cells grew to full confluence, a scratch wound was gently made in the center of the well with a 200 µL micropipette tip. The cells were washed 3 times with PBS, and the serum-free medium was added. Images were captured at 0, 6, 12, 18, and 24 h after injury. The mobility was evaluated by the width of wound healing. Quantitation of migration data was provided as % gap filling/wound healing.

### 2.14. Whole-Mount In Situ Hybridization

Whole-mount in situ hybridizations were performed as described in [18]. To generate probes, the *tyrp1b* (NM_001002749.2)*, dct* (NM_131555.2)*, tyr* (NM_131013.3) gene were PCR amplified from 24 hpf zebrafish cDNA. The fragments were cloned into pGEMT-easy vector (Promega) which was used as the template, in vitro transcribed using T7 RNA polymerase (Invitrogen). The clone primers are listed in the Supplementary Materials, Table S3.

### 2.15. Statistics

The results were all analyzed using Student’s *t*-test and presented as mean ± S.D. with a significance level of *p* < 0.05. The statistics were performed using Prism version 8.0.2 (GraphPad Software, San Diego, CA, USA). Each experiment was done at least three times.

## 3. Results

### 3.1. HTR2A Mediates the Melanogenesis Induction of Serotonin in Both B16F10 Cell Line and Zebrafish Embryos

**Figure 1:**
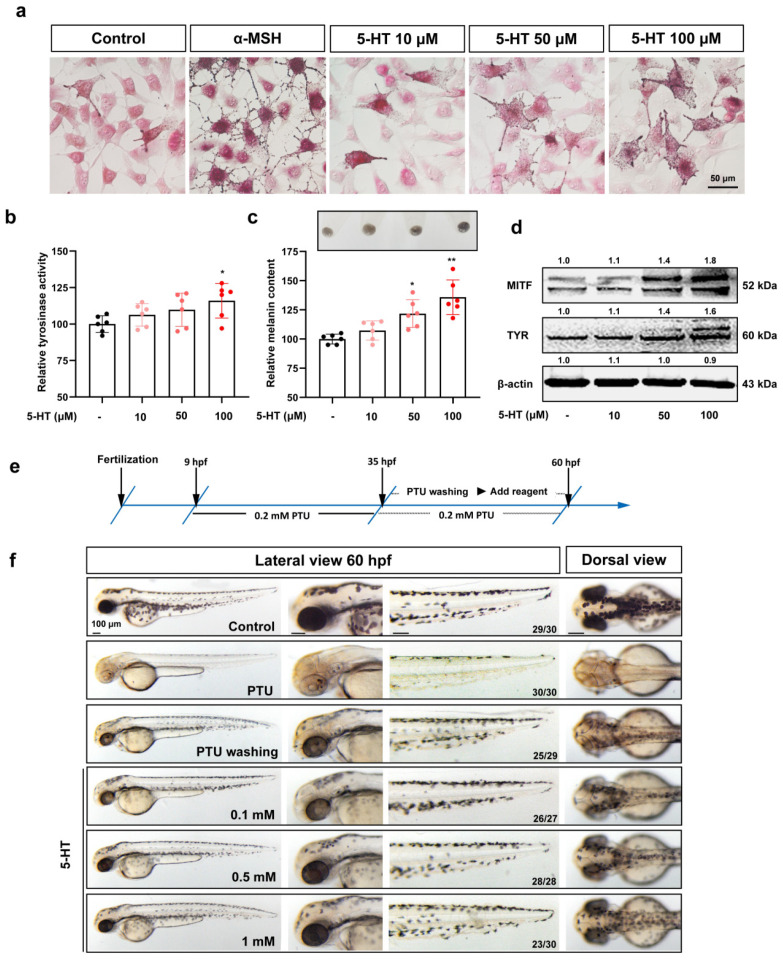
5-HT induces melanogenesis in B16F10 and zebrafish embryos: (**a**) Masson–Fonta staining of B16F10 cells to show the melanocytes morphology and melanin content with the treatment of different concentrations of 5-HT (10, 50, and 100 μM), α-MSH (50 nM) was used as a positive control. Scale bar, 50 μm; (**b**,**c**) relative tyrosinase activity (**b**) and melanin content (**c**) in B16F10 cells treated with 5-HT (10, 50, and 100 μM) (*n* = 6). The photos of melanin precipitation in the tube are at the top of the diagram (**c**); (**d**) Western blot shows the protein expression of melanin synthesis, MITF, and TYR in B16F10 cells treated with 5-HT (10, 50, and 100 μM); (**e**) the drug treatment strategy in zebrafish, 0.2 mM PTU (9–35 hpf) resulted in depigmentation in zebrafish, then 0.2 mM PTU or specific drug was added to explore the recovery of pigment in the zebrafish embryos at 60 hpf; (**f**) the morphology of melanocytes in zebrafish embryos at 60 hpf treated with 5-HT (0.1, 0.5, and 1 mM) from 35–60 hpf. The left column shows the whole view of zebrafish embryos (lateral view). The right three columns show the amplification photos of head (lateral view), trunk (lateral view), and head (dorsal view). Scale bar, 100 μm. * (*p* < 0.05) and ** (*p* < 0.01) compared to control group.

Previous studies have shown that 5-HT could promote the development of melanocytes in zebrafish embryos and the function of melanin synthesis in cultured melanocytes [21]. In this study, we confirmed the regulation of melanocyte function by 5-HT through adding excess 5-HT into the B16F10 melanoma cell line (in vitro) and PTU-treated zebrafish embryos (in vivo); α-MSH was used as a positive control. The results showed that 5-HT (10–100 μM) could dose-dependently increase tyrosinase activity, melanin content, and melanin synthesis-related protein expression in B16F10 cells (Figure 1a–d). At the same time, 5-HT could promote the pigment recovery of zebrafish embryos after PTU decolorization (Figure 1e,f). These results were consistent with previously reported research. However, serotonin receptors are a big receptor subfamily with at least 14 subtypes in mammalian, and therefore, elucidating which receptor subtype mediates 5-HT to regulate melanocyte development and function is poorly understood.

**Figure 2:**
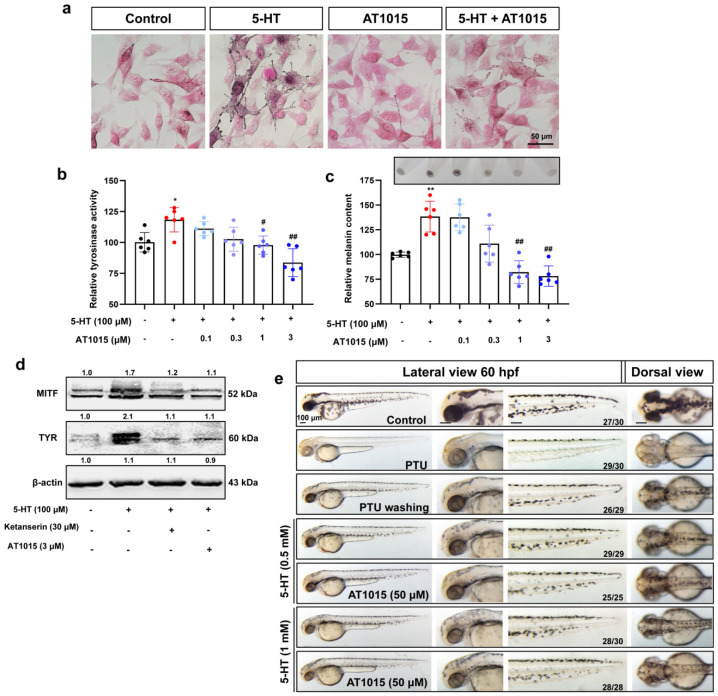
HTR2A antagonist AT1015 inhibits the melanogenesis promotion effect of 5-HT in B16F10 cells and zebrafish embryos: (**a**) Masson–Fonta staining of B16F10 cells to show the melanocytes morphology and melanin content with the treatment of 5-HT (100 μM), HTR2A antagonist AT1015 (3 μM), and cotreatment. Scale bar, 50 μm; (**b**,**c**) relative tyrosinase activity (**b**) and melanin content (**c**) in B16F10 cells treated with 5-HT (100 μM) and different concentrations of AT1015 (0.1, 0.3, 1, and 3 μM) as compared with the control (*n* = 6). The photos of melanin precipitation in the tube are at the top of the diagram (**c**); (**d**) Western blot shows the protein expression of melanin synthesis, MITF, and TYR in B16F10 cells treated with 5-HT (100 μM) or cotreated with AT1015 (3 μM) or ketanserin (30 μM); (**e**) the morphology of melanocytes in zebrafish embryos at 60 hpf. The left column shows the whole view of zebrafish embryos (lateral view). The right three columns show the amplification photos of head (lateral view), trunk (lateral view), and head (dorsal view). Scale bar, 100 μm. * (*p* < 0.05) and ** (*p* < 0.01) compared to control group; # (*p* < 0.05) and ## (*p* < 0.01) compared to 5-HT group.

Lee et al. found that 5-HT could regulate melanin production in human melanoma cells through the 5-HT2A receptor (HTR2A) [19]. This result suggests that HTR2A may play a regulatory role in melanin production. To verify that HTR2A mediates the melanin regulatory function of 5-HT, in this study, we selected AT1015 and ketanserin, two antagonists of HTR2A (Figure S1a,b). The results show that the HTR2A antagonists AT1015 (1–3 μM) (Figure 2a–c) and ketanserin (30–50 μM) (Figure S2a–c) can significantly inhibit the melanogenesis promotion effects of serotonin in B16F10 cells. Next, Western blot was used to detect the expression of related proteins MITF and TYR. The results show that 5-HT can upregulate the expression of MITF and TYR proteins. However, the upregulation of MITF and TYR proteins by 5-HT was significantly inhibited after the administration of AT1015 (3 μM) and ketanserin (30 μM) (Figure 2d).

Next, we further verified the effect of HTR2A antagonists in zebrafish. We used 5-HT (0.5 and 1 mM) and 5-HT (0.5 and 1 mM) combined with HTR2A antagonists. It was found that melanin recovered significantly with 5-HT treatment, while this effect could be inhibited by the HTR2A antagonists AT1015 (Figure 2e) and ketanserin (Figure S2d). The docking analysis showed the interaction of AT1015 and ketanserin with HTR2A protein (Figure S1e). These results further confirmed the crucial role of HTR2A in the melanogenesis induction process of serotonin.

### 3.2. The Agonists of HTR2A Promote Melanogenesis in B16F10 Cell, Cultured Human Skin Tissue, and Zebrafish Embryos

**Figure 3:**
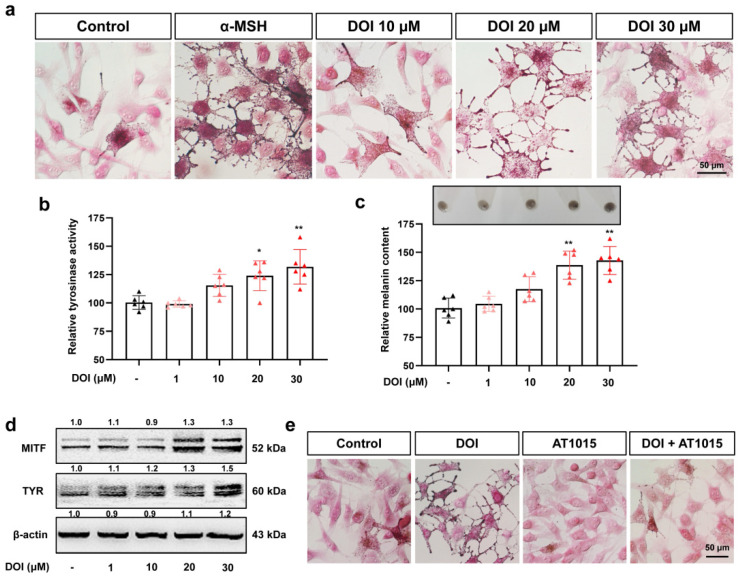
HTR2A agonist DOI induces melanogenesis in B16F10 cells: (**a**) Masson–Fonta staining of B16F10 cells treated with HTR2A agonist DOI (10, 20, and 30 μM) and α-MSH (50 nM) used as a positive control. Scale bar, 50 μm; (**b**,**c**) relative tyrosinase activity (**b**) and melanin content (**c**) in B16F10 cells treated with different concentrations of DOI (1, 10, 20, and 30 μM) as compared with the control (*n* = 6). The photos of melanin precipitation in the tube are at the top of the diagram (**c**); (**d**) Western blot shows the protein expression of melanin synthesis, MITF, and TYR in B16F10 cells treated with DOI (1, 10, 20, and 30 μM); (**e**) Masson–Fonta staining of B16F10 cells with the treatment of DOI (30 μM), HTR2A antagonist AT1015 (3 μM), and cotreatment. Scale bar, 50 μm. * (*p* < 0.05) and ** (*p* < 0.01) compared to control group.

DOI and TCB-2, which are two agonists of HTR2A, were used in this research to explore the effect of HTR2A activation in melanocyte (Figure S1c–e). Through Masson–Fonta staining of B16F10 cells, we observed more melanin deposition in the cells treated with DOI (Figure 3a) and TCB-2 (Figure S3a) in a dose dependent manner as compared with the control; α-MSH was used as a positive control. The quantitative analysis of tyrosinase activity and melanin content and the expression of MITF and TYR were consistent with these results (Figure 3b–d and Figure S3b–d). Consistent with our hypothesis, cotreatment of AT1015 inhibited the melanogenesis induction of DOI (Figure 3e) and TCB-2 (Figure S3e) in B16F10 cells.

**Figure 4:**
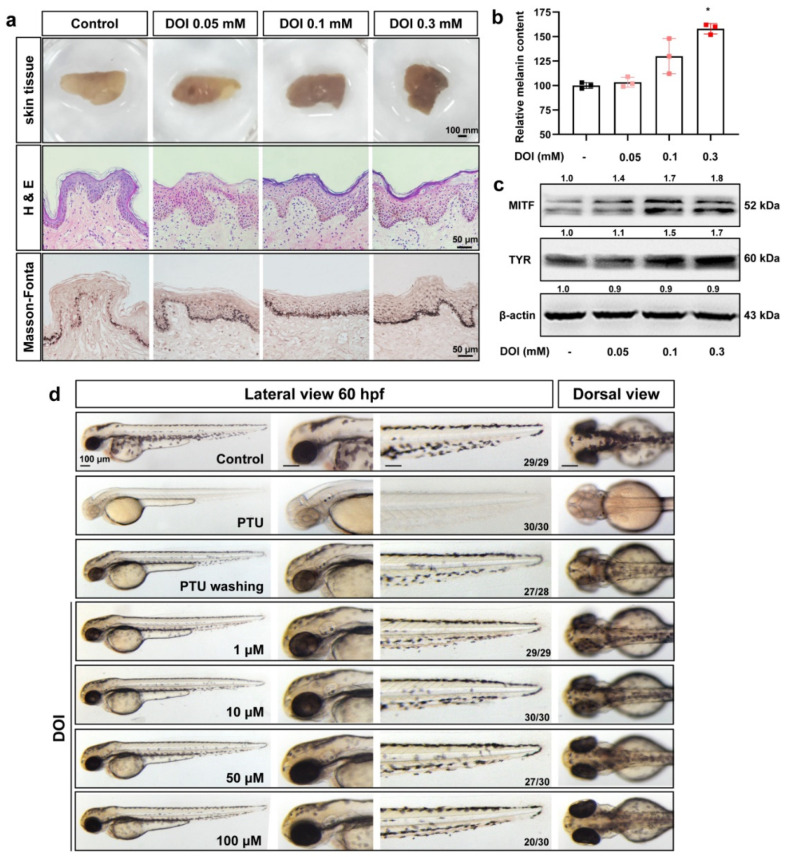
HTR2A agonist DOI increases the melanogenesis in cultured human skin tissue and zebrafish embryos. (**a**). The morphological photos, H&E staining and Masson-Fonta staining of human skin tissue treated with HTR2A agonist DOI (0.05, 0.1, and 0.3 mM). Scale bar, 100 mm (morphology of skin tissue), 50 μm (H&E staining and Masson-Fonta staining). (**b**). Relative melanin content in human skin tissue treated with DOI (0.05, 0.1, and 0.3 mM) as compared with the control (*n* = 3). (**c**). Western blot shows the protein expression of MITF and TYR in human skin tissue treated with DOI (0.05, 0.1, and 0.3 mM). (**d**). The morphology of melanocytes in zebrafish embryos at 60 hpf treated with DOI (1, 10, 50, and 100 μM) from 35–60 hpf. The left column shows the whole view of zebrafish embryos (lateral view). The right three columns show the amplification photos of head (lateral view), trunk (lateral view) and head (dorsal view). Scale bar, 100 μm.* (*p* < 0.05) compared to control group.

Given the limitation of in vitro cultured cells, we also used human skin tissue and zebrafish embryos to confirm the melanogenesis regulation of HTR2A agonists. As the results show, both DOI (Figure 4a–c) and TCB-2 (Figure S4a–c) increased the melanogenesis in cultured human skin tissue. The H&E and Masson–Fonta staining results showed that melanin synthesis was increased in the melanocytes at the basal layer of the skin with the administration of DOI or TCB-2. At the same time, more melanin was observed in the keratinocytes of the epidermis. These results indicate that HTR2A agonists can enhance the melanin synthesis in melanocytes and also increase the melanin transfer to adjacent keratinocytes. A similar increase in protein expression was observed in skin tissue. At the same time, zebrafish embryos treated with DOI (Figure 4d) or TCB-2 (Figure S4d) exhibited melanin promotion in a dose dependent manner.

### 3.3. Htr2a Deficiency Inhibits the Melanin Promotion Effect of Serotonin and HTR2A Agonist DOI in Zebrafish

**Figure 5:**
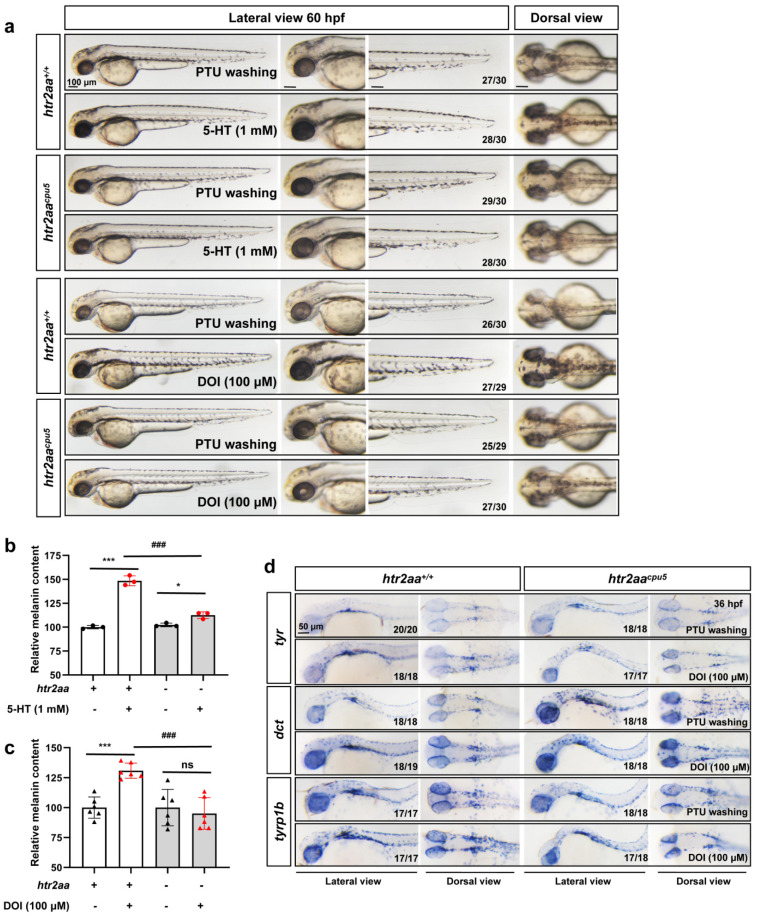
*Htr2aa* knockout in zebrafish attenuated the melanogenesis promotion of 5-HT and HTR2A agonist: (**a**) The morphology of melanocytes in zebrafish embryos at 60 hpf treated with 5-HT (1 mM) and DOI (100 μM) in wild-type siblings and *htr2aa* knockout zebrafish line *htr2aa^cpu5^* from 35–60 hpf. The left column shows the whole view of zebrafish embryos (lateral view). The right three columns show the amplification photos of head (lateral view), trunk (lateral view), and head (dorsal view). Scale bar, 100 μm; (**b**,**c**) relative melanin content in zebrafish embryos with or without *htr2aa* treated with 5-HT (**b**) (*n* = 3) or DOI (**c**) (*n* = 6); *** (*p* < 0.001) compared to control group; * (*p* < 0.05) and ns (no significance) compared to *htr2aa^−/−^* zebrafish; ### (*p* < 0.001) compared to wild type zebrafish with 5-HT or DOI treatment. (**d**) the whole mount in situ hybridization to show the expression of melanin synthesis genes, *tyr*, *dct* and *tyrp1b,* in wild-type siblings and *htr2aa* knockout zebrafish line *htr2aa^cpu5^* at 36 hpf treated with or without DOI (100 μM). Scale bar, 50 μm.

Although small-molecule inhibitors and siRNA have been commonly used in pharmacological research, the problems of selectivity of ligands and effectiveness and specificity of siRNA cannot be ignored. Therefore, we knockout the zebrafish *htr2aa* (the orthologous gene of mammalian HTR2A) using CRISPR/Cas9 to obtain two stably inherited *htr2aa* nonsense mutation zebrafish lines, namely *htr2aa^cpu5^* and *htr2aa^cpu6^* (Figure S5). Similar to the results of zebrafish embryos treated with HTR2A antagonists, AT1015 and ketanserin, genetic knockout of *htr2aa* also significantly attenuated the melanin recovery of zebrafish embryos with 5-HT or DOI treatment (Figure 5a). At the same time, *htr2aa* knockout inhibited the increase in melanin content (Figure 5b,c) and the expression of melanogenesis-related genes, *tyr*, *dct,* and *tyrp1b* (Figure 5d) with 5-HT or DOI treatment in zebrafish. This result further verified that Htr2a is the key receptor of 5-HT and DOI to regulate melanogenesis.

### 3.4. HTR2A Agonists Induce Melanosome Transport by Increasing the Melanocyte Dendrites and Migration

**Figure 6:**
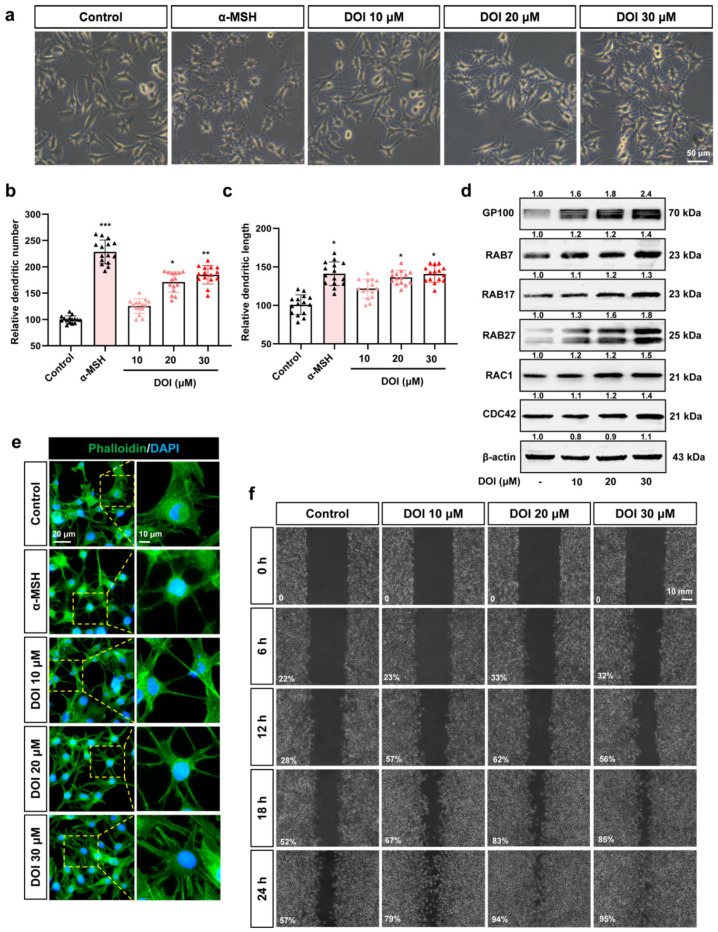
HTR2A agonist DOI induces dendrites and migration of B16F10 cells: (**a**) The cell morphology of B16F10 cells treated with different concentrations of DOI (10, 20, and 30 μM) and α-MSH (50 nM) is used as a positive control. Scale bar, 50 μm; (**b**,**c**) the dendritic number (**b**) and length (**c**) of the B16F10 cells treated with different concentrations of DOI (10, 20, and 30 μM) (*n* = 15); (**d**) Western blot shows the protein expression of GP100 (melanosome organization), RAB7/17/27 (melanosome transport), RAC1, and CDC42 (melanocytes dendrites) in B16F10 cells treated with DOI (10, 20, and 30 μM); (**e**) phalloidin staining shows the cytoskeleton in B16F10 cells treated with DOI (10, 20, and 30 μM). DAPI was used to mark the nucleus. The right row is the partial enlargement of the photos on the left. Scale bar, 20 μm (left), 10 μm (right); (**f**) cell scratch experiments show the migration of B16F10 after treatment with DOI (10, 20, and 30 μM) from 0 to 24 h. Percentage (%) of gap filling/wound healing was used to indicate the migration ability of B16F10 cells. Scale bar, 10 mm. * (*p* < 0.05), ** (*p* < 0.01) and *** (*p* < 0.001) compared to control group.

In addition to an increase in melanin synthesis, we also found that melanin transfer to karotinocytes was promoted in human skin tissue after treatment with HTR2A agonists (Figure 4a). Melanin transfer in human skin cells has been reported to be mediated by filopodia [22,23]. Recently, our research has proven that fluoxetine regulated melanocyte dendrites through HTR2A. Therefore, we assume that HTR2A takes part in the process of melanin transfer from melanocytes to keratinocytes through promoting the dendrites of melanocytes. Therefore, we used HTR2A agonists to explore the role of HTR2A in melanocyte dendrites and migration. As the results shown, dendritic number and length of B16F10 cells were increased because of DOI (Figure 6a–c) or TCB-2 (Figure S6a–c) treatment. The Western blot experiments revealed that the expression of proteins related to melanosome organization (GP100), melanosome transport (RAB7, RAB17, and RAB27), and melanocyte dendrites (RAC1 and CDC42) were increased in B16F10 cells with DOI (Figure 6d) or TCB-2 (Figure S6d) treatment [24]. Immunofluorescent staining of phalloidin showed an increase in cytoskeleton and dendrites with the DOI (Figure 6e) or TCB-2 (Figure S6e) treatment. Finally, a wound healing assays were performed to determine the migration of B16F10 cells. As expected, the B16F10 cells treated with DOI (Figure 6f) or TCB-2 (Figure S6f) showed a greater ability to migrate. These results support that HTR2A agonists promote melanin transfer through increasing the dendrites and the migration of melanocytes.

### 3.5. PKA/CREB Signaling Plays an Essential Role in the Downstream of HTR2A to Regulate Melanocyte Function

**Figure 7:**
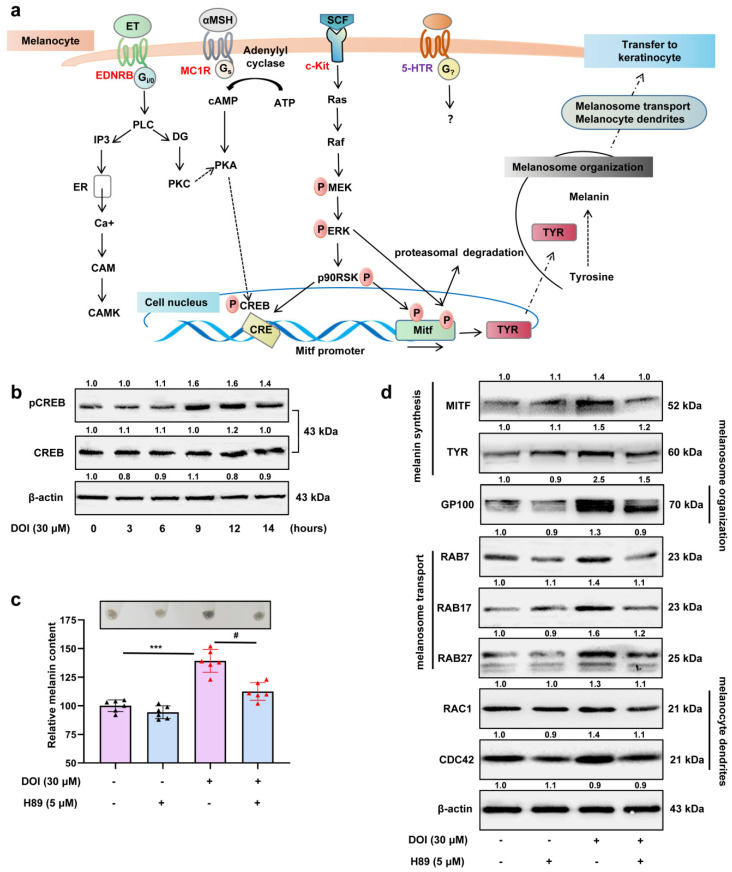
HTR2A agonist promotes melanin synthesis and melanosome transport through the PKA/CREB signaling pathway: (**a**) Schematic diagram shows the classical signals involved in the initiation of melanogenesis and the process of melanin synthesis and melanin transfer to neighboring keratinocytes; (**b**) Western blot shows the protein expression of phospho-CREB and total CREB in B16F10 cells after treatment with DOI (30 μM) from 0–14 h; (**c**) relative melanin content in B16F10 cells treated with DOI (30 μM), H89 (the inhibitor of PKA signaling, 5 μM), and cotreatment as compared with the control (*n* = 3). The photos of melanin precipitation in the tube are at the top of the diagram (**c**); (**d**) Western blot shows the protein expression of MITF and TYR (melanin synthesis), GP100 (melanosome organization), RAB7/17/27 (melanosome transport), RAC1, and CDC42 (melanocytes dendrites) in B16F10 cells treated with DOI (30 μM), H89 (5 μM), and cotreatment. *** (*p* < 0.001) compared to control group; # (*p* < 0.05) compared to the group of B16F10 cells treated with DOI.

The existing studies suggest that the HTR2A binds primarily to the G_αq_ signaling pathway, which stimulates the activation of phospholipase C (PLC) and the release of diacylglycerol (DAG) and inositol triphosphate (IP3) to stimulate protein kinase C (PKC) and Ca^2+^ release under the stimulation of receptor agonists. In addition to the G_αq_ signaling transduction pathway, HTR2A also activates a variety of molecular levels and/or activity changes such as PLA2, ERK1/2, nitric oxide, calmodulin, cAMP, AKT, Fos, and JAK/STATS. To identify the key molecular downstream mechanisms of HTR2A to regulate melanin synthesis and melanosome transport, Western blot experiments of the main signaling pathway involved in melanogenesis were performed (Figure 7a).

The results showed that the PKA/CREB (Figure 7b,c and Figure S7a–c), CAMK (Figures S8a–d and S9a–d), and ERK (Figure S10) were activated in the B16F10 cells treated with HTR2A agonists. Activation of ERK signaling can induce phosphorylation of MITF at serine-73, which subsequently leads to its proteasomal degradation, resulting in the suppression of melanin synthesis. Therefore, we hypothesized that transient activation of ERK signal may not be associated with increased melanin production. Then, small-molecule inhibitors of PKA and CAMKII were used to clarify the roles of these signaling pathways in the pigmentation induced by HTR2A activation. In order to reduce the effect of inhibition of these signaling pathways on melanin production, we selected the lowest effective dose of small-molecule antagonists for these signaling pathways. However, only the small-molecule inhibitor of PKA/CREB, H89 (5 μM), showed inhibition of melanin content and protein expressions of melanogenesis with the administration of HTR2A agonists (Figure 7c,d and Figure S7d,e). Nevertheless, the inhibitor of CAMK signaling, KN93 (1 μM), showed no effect on the melanogenesis induction of HTR2A agonists (Figures S8 and S9).

## 4. Discussion

5-HT is such an important signaling molecule that regulates a variety of physiological functions. The 5-HT receptors are an evolutionarily ancient G protein-coupled receptor superfamily (except for HTR3, which is a gated ion channel). Existing drugs that target the 5-HT signaling system are mostly focused on neurological disorders, such as depression [25,26] and migraine [27]. Fluoxetine, the classic 5-HT re-uptake inhibitor, increases 5-HT levels in the synaptic cleft and improves depression. HTR2A has been reported to be involved in the 5-HT-mediated nociceptive mechanism and has been researched as a drug target in developing peripheral antinociceptive medication [28,29]. Numerous studies have shown that multiple serotonin receptors and the enzymes related to 5-HT metabolism are expressed in skin, which are involved in the function regulation of skin cells [30]. However, studies on the role of 5-HT signaling in skin and the treatment of skin diseases by targeting 5-HT signaling system are very rare.

A reduced 5-HT level in the serum and skin of vitiligo patients and depigmentation mice with chronic unpredictable mild stress (CUMS) indicates the relationship of 5-HT level and melanocyte function [31,32,33,34]. In addition, our previous study found that fluoxetine has the effect of promoting melanocyte melanin production, which can be used in the treatment of mental-stress-induced depigmentation in mice. Nonetheless, interference of the 5-HT system in the treatment of pigment-related cutaneous diseases lacks evidentiary support.

In this study, we confirmed the role of 5-HT in promoting melanin production using the B16F10 cell line in vitro and zebrafish embryo model in vivo. We noticed that multiple studies have indicated that HTR2A may be the key receptor subtype mediating the melanin promoting effect of 5-HT signal. At the same time, we found that HTR2A agonists can promote melanin production in cultured melanocytes and also in human skin tissues and zebrafish embryos at lower concentrations than 5-HT. The antagonists of HTR2A (AT1015 or ketanserin) could block the melanogenesis promotion effect of 5-HT or the HTR2A agonists (DOI or TCB-2). These results suggest that HTR2A agonists may have potential applications in the treatment of skin diseases with abnormal melanin metabolism. In particular, simultaneous analysis of multiple signal transduction mechanisms in cell culture systems has shown that the same ligand may have differing degrees of intrinsic activity for the activation of different second messenger systems by the same population of HTR2A receptors [35]. On consideration of the selectivity of small-molecule ligands, we used two antagonists and two agonists in parallell to confirm the key role of HTR2A in regulating melanocyte function. Additionally, when we genetically deleted Htr2a in zebrafish, the melanin promoting effect of 5-HT and DOI was significantly inhibited. These results suggest that HTR2A has certain specificity in mediating the melanin promoting effect of its agonist.

Interestingly, we found that neither HTR2A antagonist nor *htr2aa* knockout affected melanocyte development and melanin production in zebrafish embryos (data not shown). We presume that HTR2A is not essential for the normal survival or maintenance of physiological functions of melanocytes under physiological conditions. However, another possibility is that *htr2ab* (another orthologus gene of *HTR2A* in zebrafish) may partially compensate for the function of *htr2aa*. Therefore, only deletion of *htr2aa* is not sufficient to affect melanocyte survival or function.

In addition, we noted that HTR2A receptor agonists significantly increased the dendrites and migration of B16F10 cell in vitro. At the same time, we also observed an increase in the number of melanin particles in epidermal keratinocytes in cultured human skin. These discoveries further confirmed the positive role of HTR2A in regulating melanin transport. However, due to species differences, there is no transport process of melanin from melanocytes to keratinocytes in zebrafish models. Therefore, no effect of 5-HT or HTR2A agonists on melanin transport was observed in the zebrafish experiments. Furthermore, B16F10 cell is the mouse melanoma model which is commonly used in oncological research. High metastasis and invasiveness are the main reasons why melanoma is difficult to cure. Due to the regulation on the cell migration of HTR2A ligands, we speculate that inhibition of HTR2A may be a new idea to prevent metastasis of melanoma cells.

Finally, we identified the PKA/CREB signaling act downstream of HTR2A to regulate melanin synthesis and melanosome transport. Although the role of 5-HT/HTR2A has already been reported, the downstream molecular mechanisms are not completely understood. Multiple signaling pathways involve in the progress of melanogenesis, such as PKA/CREB, CAMKII, and MAPK. These signals act in coordination, triggering a cascade of reactions that guarantee homeostasis in human bodies. Here, we found that PKA signaling may act downstream of 5-HT/HTR2A to regulate melanin production through small-molecule inhibitors. At the same time, we also noticed that HTR2A is an established activator of the G_αq_ signaling cascade that leads IP3 generation and the release of Ca^2+^ from the endoplasmic reticulum. The release of Ca^2+^ from the ER and subsequent activation of the ER Ca^2+^ sensor (STIM1, stromal interaction molecule 1) also play a critical role in melanogenesis. Therefore, it would be worthwhile to investigate the role of this signal in 5-HT/HTR2A-induced melanin production in the future. Taken together, the 5-HT/HTR2A-PKA/CREB signal axis plays an important role in regulating melanin synthesis and transfer. Furthermore, targeting HTR2A is a potential method to develop drugs for skin diseases with melanin metabolism disorder.

## Data Availability

The data presented in this study are available in article and Supplementary Materials.

## Supplementary Materials

The following supporting information can be downloaded at https://www.mdpi.com/1422-0067/23/11/6111/s1.

## Abbreviations and Nomenclature

5-HT	serotonin (5-hydroxytryptamine)
HTR2A	serotonin receptor 2A
DOI	4-iodo-2,5-dimethoxy-α-methylbenzeneethanamine hydrochloride
TCB-2	4-bromo-3,6-dimethoxybenzocyclobuten-1-yl methylamine hydrobromide
ketanserin tartrate	3-[2-[4-(4-fluorobenzoyl)-1-piperidinyl]ethyl]-2,4[1H,3H]-quinazolinedione tartrate
AT1015	N-[2-[4-(5H-dibenzo[a,d]cyclohepten-5-ylidene)-1-piperidinyl]ethyl]-1-formyl-4-piperidinecarboxamide hydrochloride
NE	Norepinephrine
EP	Epinephrine
GABA	gamma-aminobutyric acid
HA	histamine
GPCR	G protein-coupled receptors
DMEM	Dulbecco’s modified Eagle’s medium
FBS	fetal bovine serum
sgRNA	single guide RNA
PTU	phenylthiourea
siRNA	small interference RNA
PLC	phospholipase C
DAG	diacylglycerol
IP3	inositol triphosphate
PKC	protein kinase C
PKA	protein kinase A
hpf	hours post fertilization
