# Supplementary material for: Serotonin (5-HT) 2A Receptor Involvement in Melanin Synthesis and Transfer via Activating the PKA/CREB Signaling Pathway
Source: Int J Mol Sci. 2022 May 30;23(11):6111. doi: 10.3390/ijms23116111 (PMC9245606; doi:10.3390/ijms23116111)

Supplemental Materials

Supplemental Table S1. Chemicals used in this research.

Chemicals	Stock concentration	Supplier
5-HT	1 M	Sigma
α-MSH	50 μM	Sigma
AT1015	100 mM	APExBIO
Ketanserin	100 mM	MedChemExpress
DOI	100 mM	Sigma
TCB-2	100 mM	APExBIO
PTU	20 mM	Sigma
H89	10 mM	MedChemExpress
KN93	1 mM	MedChemExpress
PD98059	20 mM	Cell signaling technology
Phalloidin	200 μg/mL	Sigma

Supplemental Table S2. The antibody used in this research.

Protein	Antibody supplier	Cat log.
MITF	Abcam	ab20663
TYR	Abcam	ab180753
GP100	Abcam	ab137078
RAB7	Cell signaling technology	8013
RAB17	Cell signaling technology	9367
RAB27	Cell signaling technology	69295
RAC1	Abcam	ab155938
CDC42	Abcam	ab187643
CREB	Cell signaling technology	9197
P-CREB (Ser133)	Cell signaling technology	9198
CAMKII-γ	Abcam	ab37999
p-CAMKII-γ(T286)	Abcam	ab32678
p38	Cell signaling technology	8690
P-p38 (Thr180/Tyr182)	Cell signaling technology	4511
ERK1/2	Cell signaling technology	4695
P-ERK1/2 (Thr202/Tyr204)	Cell signaling technology	4370
JNK	Cell signaling technology	9258
P-JNK (Thr183/Tyr185)	Cell signaling technology	4668
β-actin	Santa cruze	sc-69879
Goat Anti-Mouse IgG H&L (HRP)	Abcam	ab6789
Goat Anti-Rat IgG H&L(Alexa Fluor® 405)	Abcam	ab175671

Supplemental Table S3. The oligo sequences used in this research.

Gene name	Species	Forward primer	Reverse primer
tyr clone	*Danio. Rerio*	AGGGTTCTGTCAGGACGTC	GACTCTACATCGGCGGATG
dct clone	*Danio. Rerio*	AGCGCTTTGTGTTGATGCTG	TGGTGCCGTTACACAGAGTC
tyrp1b clone	*Danio. Rerio*	GGAAGAGTGTGTGTTTAG-TGC	AATCTTTGAACCATT-GGACGG
htr2aa sgRNA-1	*Danio. Rerio*	ACATGGGACACAGTGATGCA**GGG**	
htr2aa sgRNA-2	*Danio. Rerio*	AATTGCACACAGGTGCATGA**TGG**	
htr2aa sgRNA-3	*Danio. Rerio*	TCTTGGCCCGGGCTCGGGAG**CGG**	
htr2aa se-quencing	*Danio. Rerio*	TCATGCCTTCCCGTGAAATC	ATTCACTCACTGCCTCACTC

Supplemental Figure legends

Supplemental Figure S1. The HTR2A agonists (DOI, TCB-2) and antagonists (Ketanserin, AT1015). a-d. MTT assay to determine the cytotoxicity dosage of AT1015 (a), Ketanserin (b), DOI (c), TCB-2 (d) in B16F10 cells. e. Docking analysis of HTR2A protein with the four ligands.

Supplemental Figure S2. HTR2A antagonists, Ketanserin inhibits the melanogenesis promotion effect of 5-HT in B16F10 cells and zebrafish embryos. a. Masson-Fonta staining of B16F10 cells to show the melanocytes morphology and melanin content with the treatment of 5-HT (100 μM), HTR2A antagonist ketanserin (30 μM), and co-treatment. Scale bar, 50 μm. b-c. Relative tyrosinase activity (b) and melanin content (c) in B16F10 cells treated with 5-HT (100 μM) and different concentration ketanserin (1, 10, 30, 50 μM) compared with control. The photos of melanin precipitation in the tube were on the top of the diagram (c). d. The morphology of melanocytes in zebrafish embryos at 60 hpf. The left coloumn shows the whole view of zebrafish embryos (lateral view). The right three coloumns show the amplification photos of head (lateral view), trunk (lateral view) and head (dorsal view). Scale bar, 100 μm.

Supplemental Figure S3. HTR2A agonist TCB-2 induces melanogenesis in B16F10 cells.

a. Masson-Fonta staining of B16F10 cells treated with HTR2A agonist TCB-2 (1, 3, 10 μM). α-MSH (50 nM) used as a positive control. Scale bar, 50 μm. b-c. Relative tyrosinase activity (b) and melanin content (c) in B16F10 cells treated with different concentration TCB-2 (1, 3, 10 μM) compared with control. The photos of melanin precipitation in the tube were on the top of the diagram (c). d. Western blot shows the protein expression of melanin synthesis, MITF and TYR in B16F10 cells treated with TCB-2 (1, 3, 10 μM). e. Masson-Fonta staining of B16F10 cells with the treatment of TCB-2 (10 μM), HTR2A antagonist AT1015 (3 μM), and co-treatment. Scale bar, 50 μm.

Supplemental Figure S4. HTR2A agonist TCB-2 increases the melanogenesis in cultured human skin tissue and zebrafish embryos.

a. The morphological photos, H&E staining and Masson-Fonta staining of human skin tissue treated with HTR2A agonist TCB-2 (30, 50, 100 μM). Scale bar, 100 mm (morphology of skin tissue), 50 μm (H&E staining and Masson-Fonta staining). b. Relative melanin content in human skin tissue treated with TCB-2 (30, 50, 100 μM) compared with control. c. Western blot shows the protein expression of MITF and TYR in human skin tissue treated with TCB-2 (30, 50, 100 μM). d. The morphology of melanocytes in zebrafish embryos at 60 hpf treated with TCB-2(1, 10, 30, 50 μM) from 35-60 hpf. The left coloumn shows the whole

view of zebrafish embryos (lateral view). The right three coloumns show the amplification photos of head (lateral view), trunk (lateral view) and head (dorsal view). Scale bar, 100 μm.

Supplemental Figure S5. The establishment of zebrafish *htr2aa* knockout line, *htr2aa*^{cpu5} and *htr2aa*^{cpu6}.

a. The genome structure of zebrafish htr2aa and the sequence of sgRNA target (green). The red base sequence indicates the PAM. b. The genome and protein sequence of the two htr2aa mutant line, *htr2aa*^{cpu5} and *htr2aa*^{cpu6}.

Supplemental Figure S6. HTR2A agonist TCB-2 induces dendrites and migration of B16F10 cells.

a. The cell morphology of B16F10 cells treated with different concentration TCB-2 (1, 3, 10 μM). α-MSH (50 nM) used as a positive control. Scale bar, 50 μm. b-c The dendritic number (b) and length (c) of the B16F10 cells treated with different concentration TCB-2 (1, 3, 10 μM). d. Western blot shows the protein expression of GP100 (melanosome organization), RAB7/17/27 (melanosome transport), RAC1 and CDC42 (melanocytes dendrites) in B16F10 cells treated with TCB-2 (1, 3, 10 μM). e. Phalloidin staining shows the cytoskeleton in B16F10 cells treated with TCB-2 (1, 3, 10 μM). DAPI used to mark the nucleus. The right row is the partial enlargement of the photos on the left. Scale bar, 20 μm (left), 10 μm (right). f. Cell scratch experiments show the migration of B16F10 after treated with TCB-2 (1, 3, 10 μM) from 0-24 hours. Scale bar, 10 mm.

Supplemental Figure S7. PKA/CREB signaling pathway mediates the melanogenesis promotion effect of HTR2A agonist TCB-2.

a-b. The relative protein expression of phospho-CREB in B16F10 cells treated with DOI (30 μM) or TCB-2 (10 μM). c.Western blot shows the protein expression of phospho-CREB and total CREB in B16F10 cells after treated with TCB-2 (10 μM) from 0-14 hours. d. Relative melanin content in B16F10 cells treated with TCB-2 (10 μM), H89 (the inhibitor of PKA signaling, 5 μM) and co-treatment compared with control. The photos of melanin precipitation in the tube were on the top of the diagram. e. Western blot shows the protein expression of MITF and TYR (melanin synthesis), GP100 (melanosome organization), RAB7/17/27 (melanosome transport), RAC1 and CDC42 (melanocytes dendrites) in B16F10 cells treated with TCB-2 (10 μM), H89 (5 μM) and co-treatment.

Supplemental Figure S8-9. CAMK signaling is not the key downstream to regulate melanogenesis of HTR2A agonist DOI (Figure S8) and TCB-2 (Figure S9) in B16F10 cells.

a. Western blot shows the protein expression of phospho-CAMKII and total CAMKII-γ in B16F10 cells after treated with DOI (10, 20, 30 μM) (Figure S8 a) or TCB-2 (1, 3, 10 μM) (Figure S9 a). b. Western blot shows the protein expression of phospho-CAMKII and total CAMKII-γ in B16F10 cells after treated with DOI (30 μM) (Figure S8 b) or TCB-2 (10 μM) (Figure S9 b) from 0-120 minutes. c-d. The quantitative analysis of protein expression in Figure S8 c-d and Figure S9 c-d. e. Relative melanin content in B16F10 cells treated with DOI (30 μM) (Figure S8 e) or TCB-2 (10 μM) (Figure S9 e), KN93 (the inhibitor of CAMK signaling, 1 μM) and co-treatment compared with control. The photos of melanin precipitation in the tube were on the top of the diagram. f. Western blot shows the protein expression of MITF and TYR (melanin synthesis), GP100 (melanosome organization), RAB7/17/27 (melanosome transport), RAC1 and CDC42 (melanocytes dendrites) in B16F10 cells treated with DOI (30 μM) (Figure S8 f) or TCB-2 (10 μM) (Figure S9 f) and KN93 (1 μM) or co-treatment.

Supplemental Figure S10. ERK signaling is transient activated in B16F10 cell with HTR2A agonist DOI and TCB-2. a and c. Western blot shows the protein expression of phospho-p38, phospho-ERK1/2, phospho-JNK and total p38, ERK1/2, JNK in B16F10 cells after treated with DOI (30 μM) (a) or TCB-2 (10 μM) (c) from 0-120 minutes. b and d. The quantitative analysis of protein expression.

Supplemental Figure S12-14. The quantitative analysis of protein expression.

Figure S1

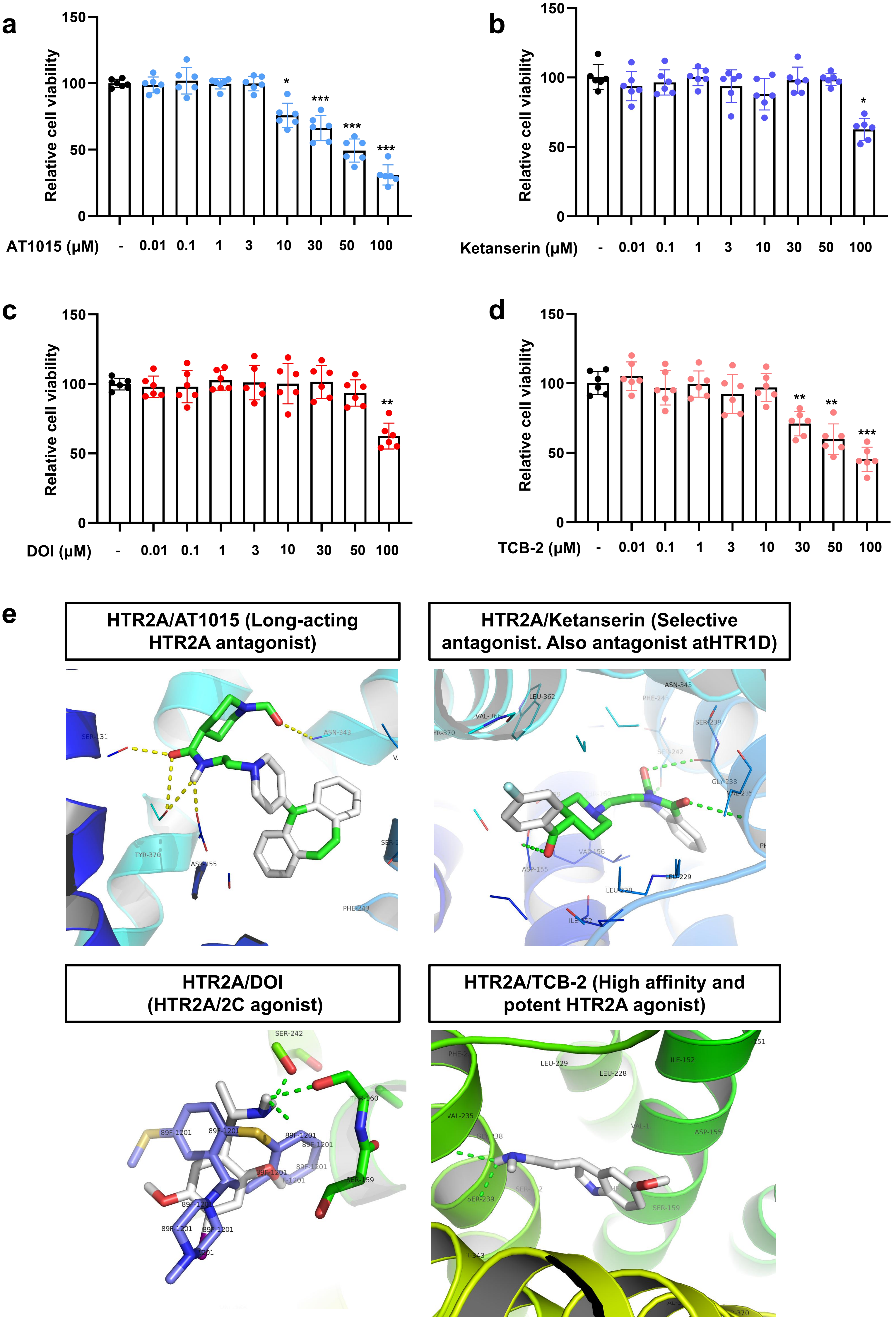

a
Relative cell viability
AT1015 (μM)
- 0.01 0.1 1 3 10 30 50 100
b
Relative cell viability
Ketanserin (μM)
- 0.01 0.1 1 3 10 30 50 100
c
Relative cell viability
DOI (μM)
- 0.01 0.1 1 3 10 30 50 100
d
Relative cell viability
TCB-2 (μM)
- 0.01 0.1 1 3 10 30 50 100
e
HTR2A/AT1015 (Long-acting HTR2A antagonist)
HTR2A/Ketanserin (Selective antagonist. Also antagonist atHTR1D)
HTR2A/DOI (HTR2A/2C agonist)
HTR2A/TCB-2 (High affinity and potent HTR2A agonist)

Figure S2

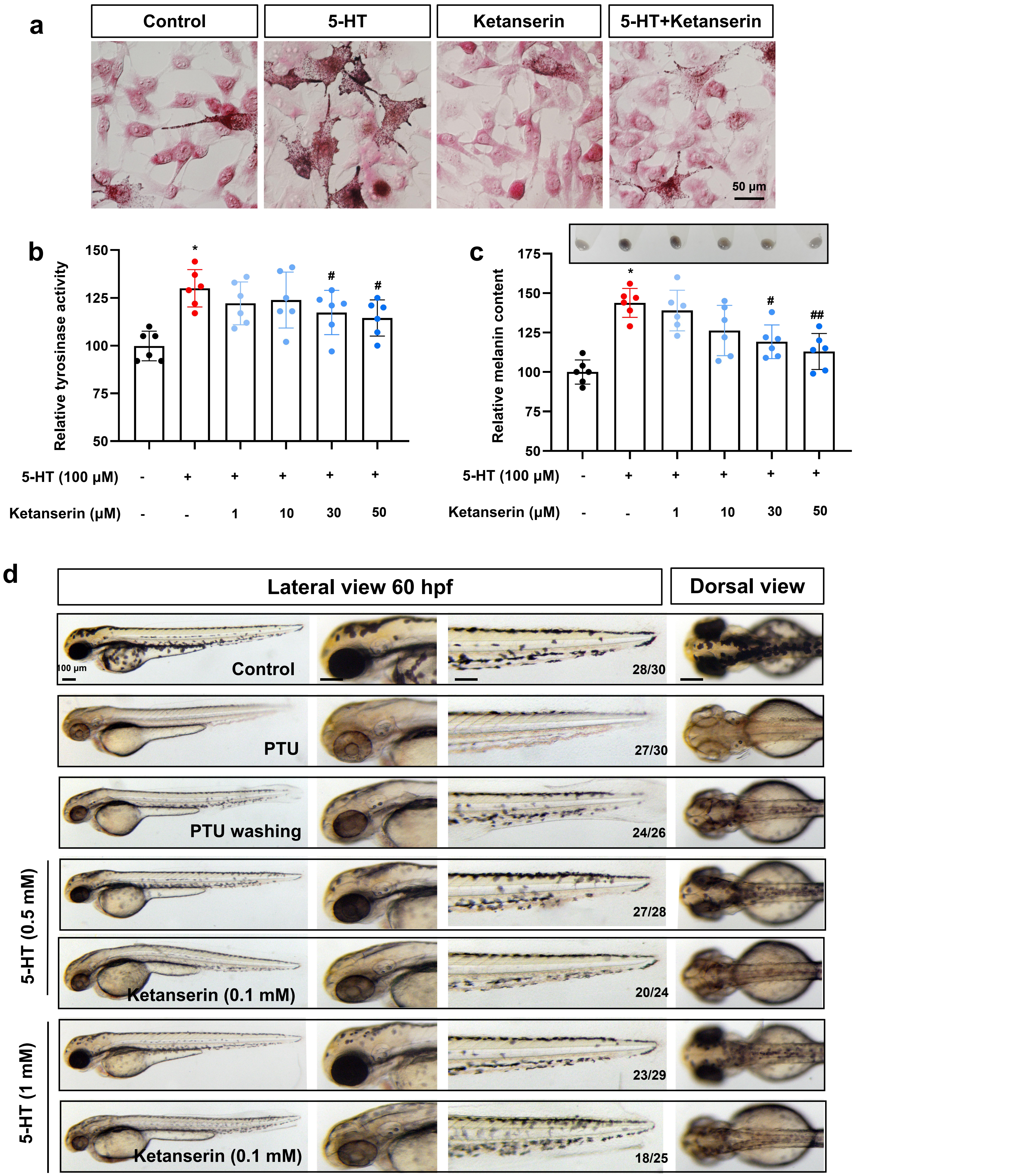
a
Control
5-HT
Ketanserin
5-HT+Ketanserin
50 μm
b
Relative tyrosinase activity
150
125
100
75
50
*
#
#
5-HT (100 μM) - + + + + +
Ketanserin (μM) - - 1 10 30 50
c
Relative melanin content
175
150
125
100
75
50
*
#
##
5-HT (100 μM) - + + + + +
Ketanserin (μM) - - 1 10 30 50
d
Lateral view 60 hpf
Dorsal view
100 μm
Control
28/30
PTU
27/30
PTU washing
24/26
5-HT (0.5 mM)
27/28
Ketanserin (0.1 mM)
20/24
5-HT (1 mM)
23/29
Ketanserin (0.1 mM)
18/25

Figure S3

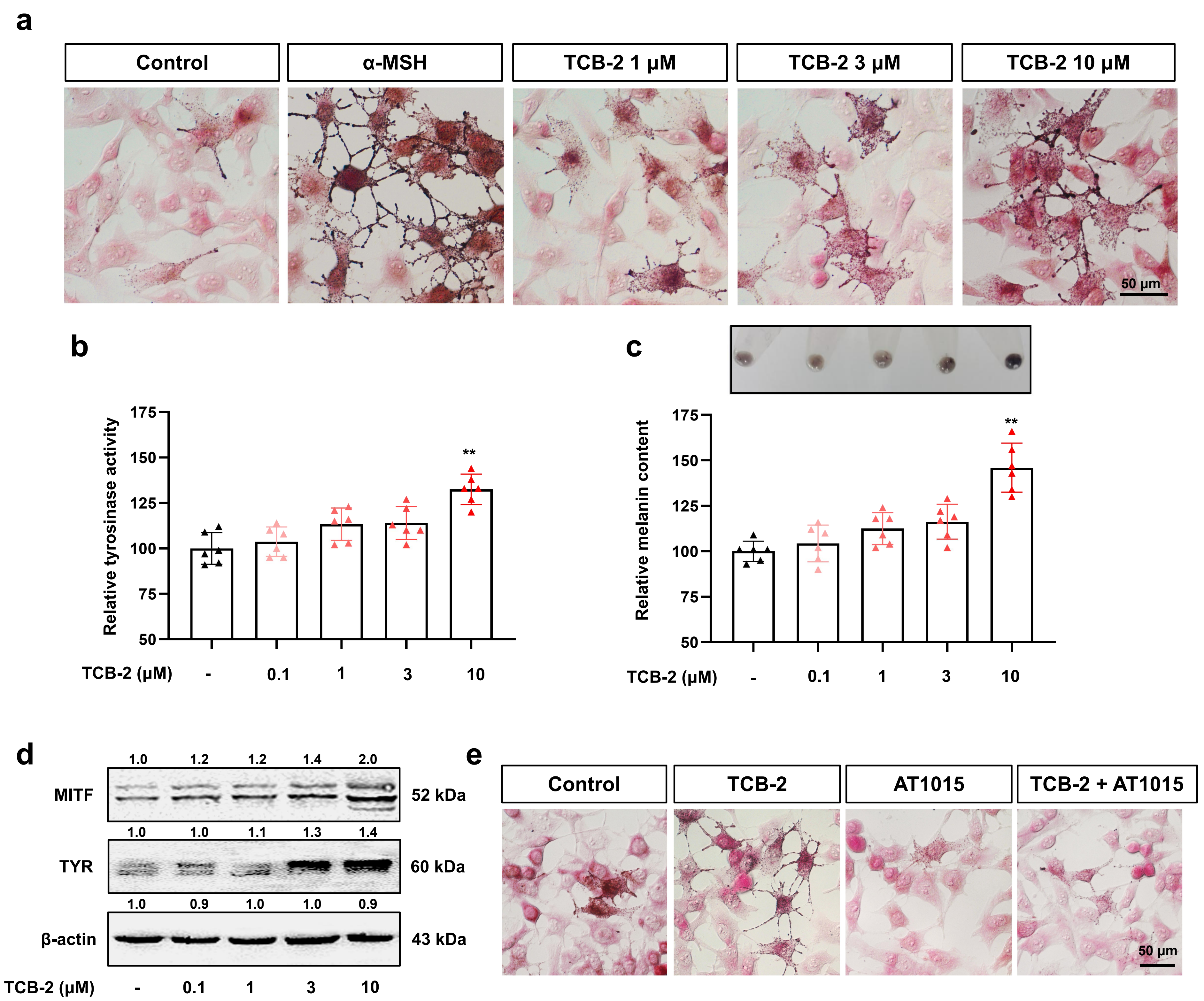

a
Control
α-MSH
TCB-2 1 μM
TCB-2 3 μM
TCB-2 10 μM
50 μm
b
Relative tyrosinase activity
175
150
125
100
75
50
**
TCB-2 (μM)
-
0.1
1
3
10
c
Relative melanin content
175
150
125
100
75
50
**
TCB-2 (μM)
-
0.1
1
3
10
d
1.0 1.2 1.2 1.4 2.0
MITF
52 kDa
1.0 1.0 1.1 1.3 1.4
TYR
60 kDa
1.0 0.9 1.0 1.0 0.9
β-actin
43 kDa
TCB-2 (μM)
-
0.1
1
3
10
e
Control
TCB-2
AT1015
TCB-2 + AT1015
50 μm

Figure S4

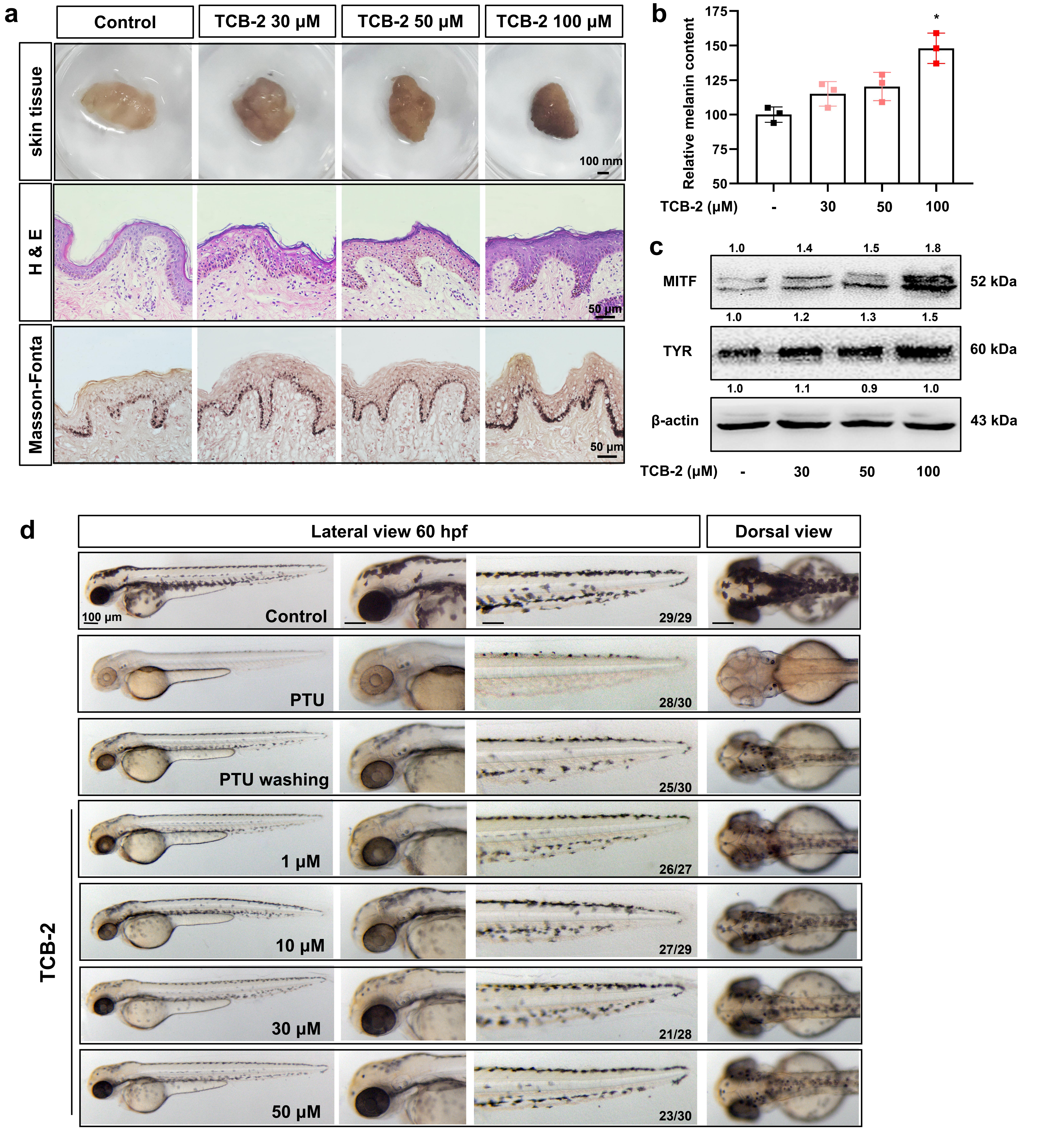
a
Control
TCB-2 30 μM
TCB-2 50 μM
TCB-2 100 μM
skin tissue
H & E
Masson-Fonta
100 mm
50 μm
50 μm
b
Relative melanin content
175
150
125
100
75
50
*
TCB-2 (μM)
-
30
50
100
c
1.0 1.4 1.5 1.8
MITF
52 kDa
1.0 1.2 1.3 1.5
TYR
60 kDa
1.0 1.1 0.9 1.0
β-actin
43 kDa
TCB-2 (μM) - 30 50 100
d
Lateral view 60 hpf
Dorsal view
100 μm
Control
29/29
PTU
28/30
PTU washing
25/30
TCB-2
1 μM
26/27
10 μM
27/29
30 μM
21/28
50 μM
23/30

Figure S5

a

zebrafish *htr2aa* genome

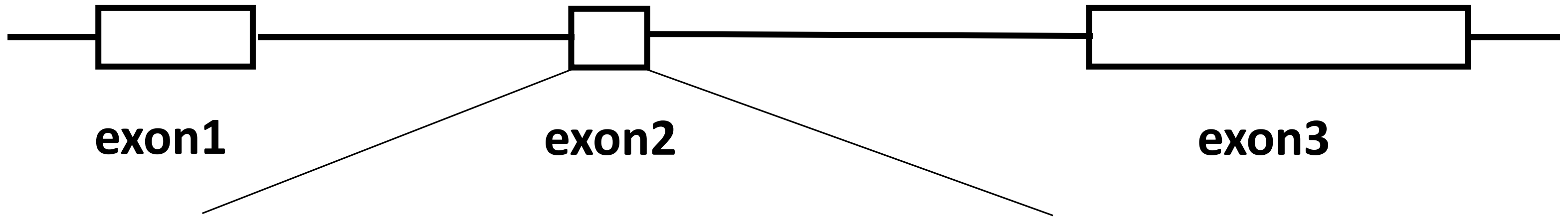

GTTACTCATGGCCTTTCCCTGCATCACTGTGTCCCATGTGGATCTACCTAGATGTGCTCTTC
TCCACCGCCTCCATCATGCACCTGTGTGCAATTTCTCTTGACCGCTATGTGGCCATTCGTAA
CCCCATCCGACACAATCGGTCAAATTCCCGCTCCCGAGCCCGGGCCAAGATTACGGCAGTCT
GGACCATCTCTGCAG

b

htr2aa$^{+/+}$ TCATGGCCTTTCCCTGCATCACTGTGTCCCATGTGGATCTACCTAGA

htr2aa^{cpu5} TCATGGCCTTTC--------ACTGTGTCCCATGTGGATCTACCTAGA △8 bp

htr2aa^{cpu6} TCATGGCCTTTCCCTG--TCACTGTGTCCCATGTGGATCTACCTAGA △2+10 bp
TGTCCCTGTG

Htr2aa$^{+/+}$ DMLLGLLVMPVSMVTIVYGYSWPFPASLCPMWIYLDVLF---------* 491 aa

Htr2aa^{cpu5} DMLLGLLVMPVSMVTIVYGYSWPFTVSHVDLPR-----* 163 aa

Htr2aa^{cpu6} DMLLGLLVMPVSMVTIVYGYSWPFPVSLCHCVPCGST* 162 aa

Figure S6

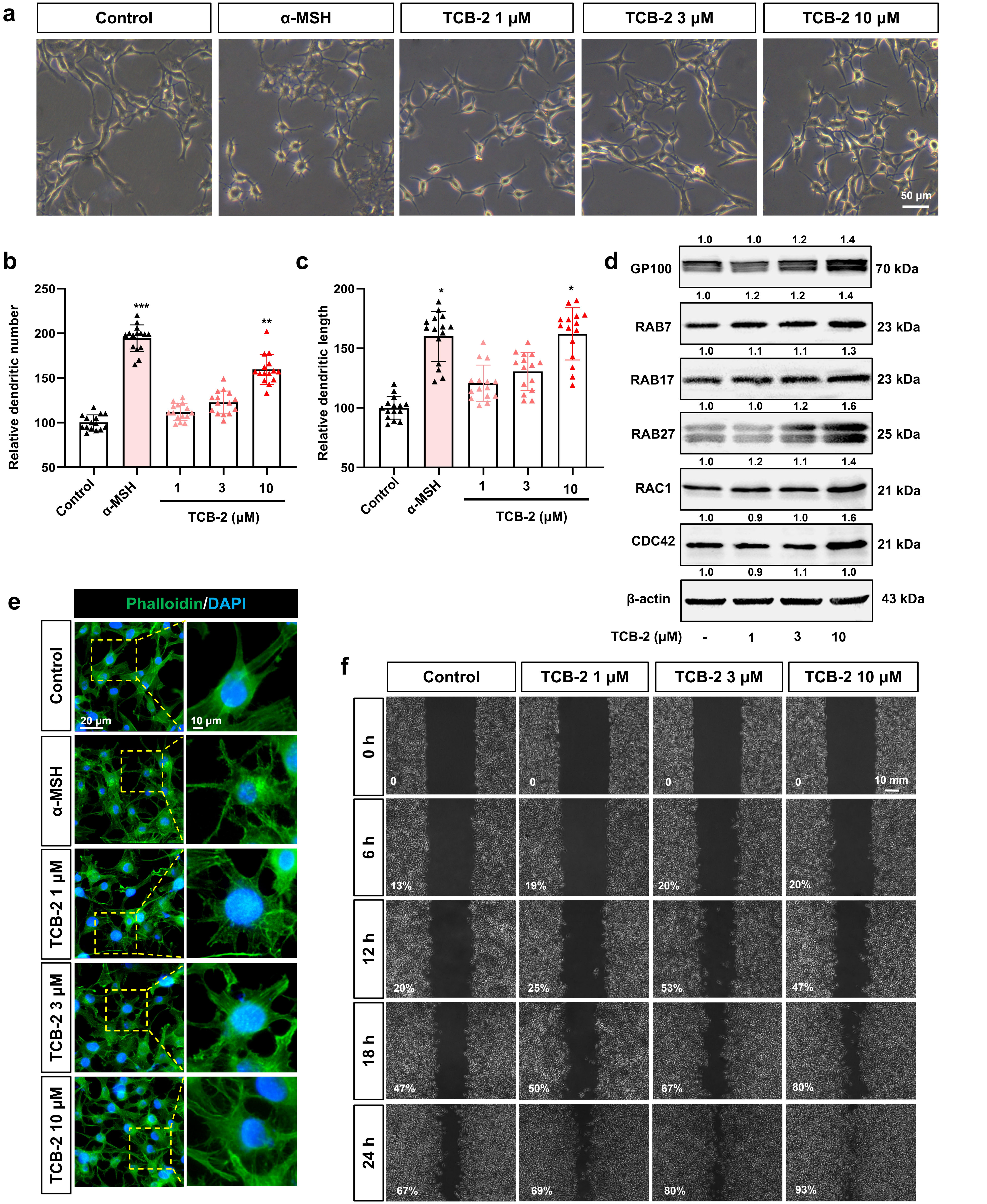
a
Control
α-MSH
TCB-2 1 μM
TCB-2 3 μM
TCB-2 10 μM
50 μm
b
Relative dendritic number
250
200
150
100
50
***
**
Control
α-MSH
1
3
10
TCB-2 (μM)
c
Relative dendritic length
200
150
100
50
*
*
Control
α-MSH
1
3
10
TCB-2 (μM)
d
GP100
1.0 1.0 1.2 1.4
70 kDa
RAB7
1.0 1.2 1.2 1.4
23 kDa
RAB17
1.0 1.1 1.1 1.3
23 kDa
RAB27
1.0 1.0 1.2 1.6
25 kDa
RAC1
1.0 1.2 1.1 1.4
21 kDa
CDC42
1.0 0.9 1.0 1.6
21 kDa
β-actin
1.0 0.9 1.1 1.0
43 kDa
TCB-2 (μM) - 1 3 10
e
Phalloidin/DAPI
Control
20 μm
10 μm
α-MSH
TCB-2 1 μM
TCB-2 3 μM
TCB-2 10 μM
f
Control
TCB-2 1 μM
TCB-2 3 μM
TCB-2 10 μM
0 h
0 0 0 0
10 mm
6 h
13% 19% 20% 20%
12 h
20% 25% 53% 47%
18 h
47% 50% 67% 80%
24 h
67% 69% 80% 93%

Figure S7

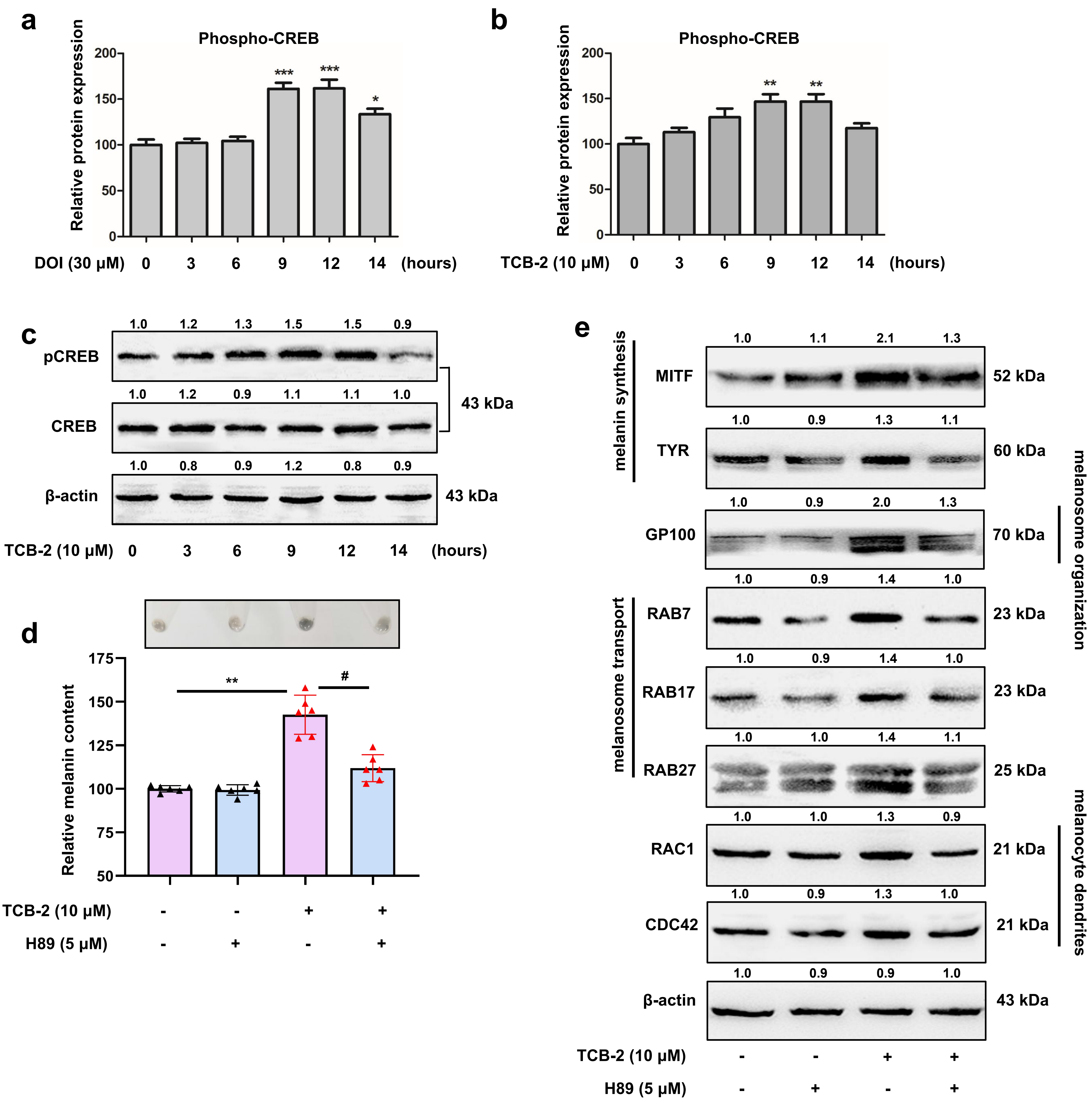

a
Phospho-CREB
Relative protein expression
200
150
100
50
0
***
***
*
DOI (30 μM) 0 3 6 9 12 14 (hours)
b
Phospho-CREB
Relative protein expression
**
**
TCB-2 (10 μM) 0 3 6 9 12 14 (hours)
c
pCREB
1.0 1.2 1.3 1.5 1.5 0.9
CREB
1.0 1.2 0.9 1.1 1.1 1.0
43 kDa
β-actin
1.0 0.8 0.9 1.2 0.8 0.9
43 kDa
TCB-2 (10 μM) 0 3 6 9 12 14 (hours)
d
Relative melanin content
175
150
125
100
75
50
**
#
TCB-2 (10 μM) - - + +
H89 (5 μM) - + - +
e
melanin synthesis
MITF 1.0 1.1 2.1 1.3 52 kDa
TYR 1.0 0.9 1.3 1.1 60 kDa
GP100 1.0 0.9 2.0 1.3 70 kDa
melanosome organization
melanosome transport
RAB7 1.0 0.9 1.4 1.0 23 kDa
RAB17 1.0 0.9 1.4 1.0 23 kDa
RAB27 1.0 1.0 1.4 1.1 25 kDa
melanocyte dendrites
RAC1 1.0 1.0 1.3 0.9 21 kDa
CDC42 1.0 0.9 1.3 1.0 21 kDa
β-actin 1.0 0.9 0.9 1.0 43 kDa
TCB-2 (10 μM) - - + +
H89 (5 μM) - + - +

Figure S8

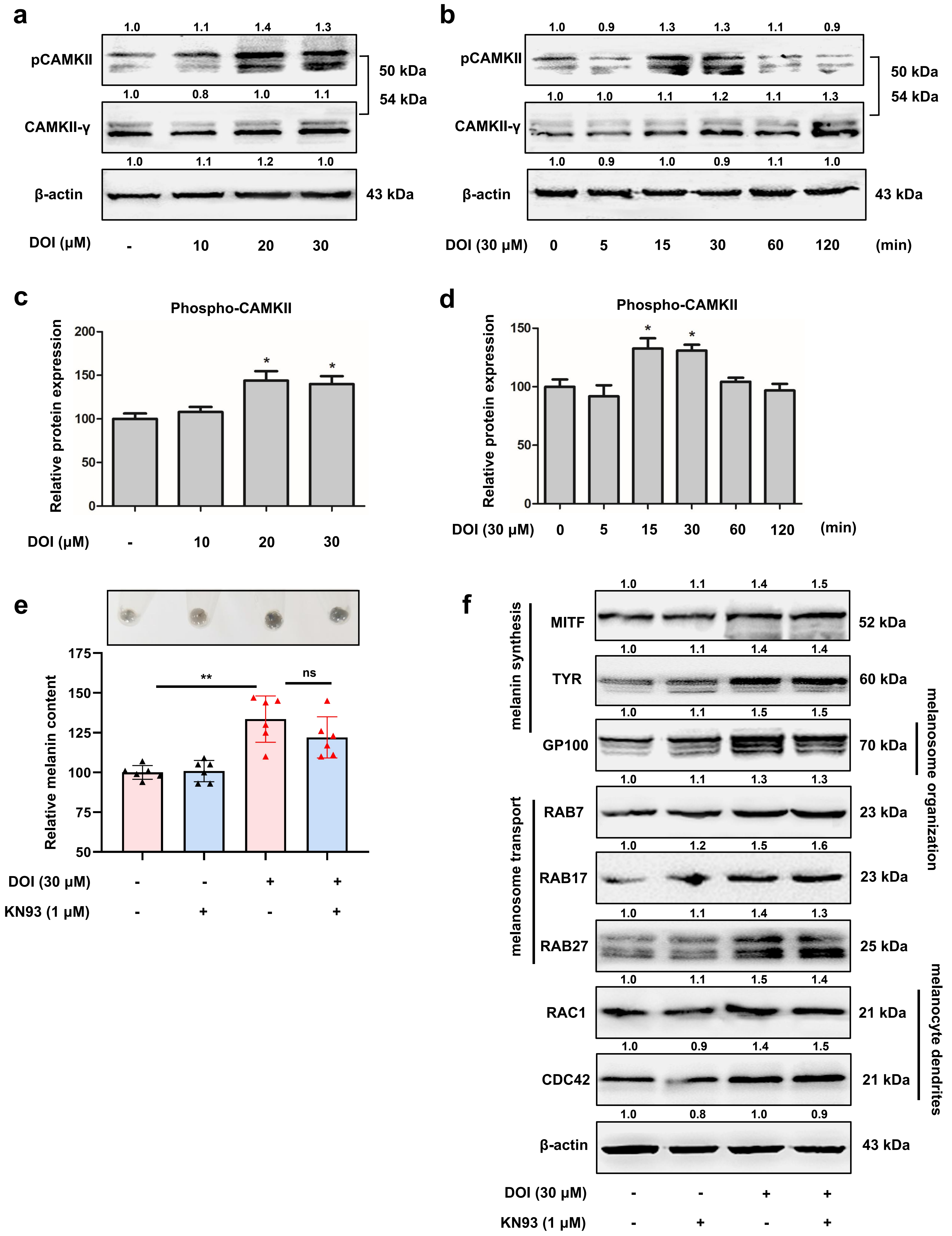

a
pCAMKII
CAMKII-γ
β-actin
50 kDa
54 kDa
43 kDa
DOI (μM) - 10 20 30
b
DOI (30 μM) 0 5 15 30 60 120 (min)
c
Phospho-CAMKII
Relative protein expression
d
Phospho-CAMKII
e
Relative melanin content
DOI (30 μM)
KN93 (1 μM)
f
MITF
TYR
GP100
RAB7
RAB17
RAB27
RAC1
CDC42
β-actin
52 kDa
60 kDa
70 kDa
23 kDa
25 kDa
21 kDa
melanin synthesis
melanosome transport
melanosome organization
melanocyte dendrites

Figure S9

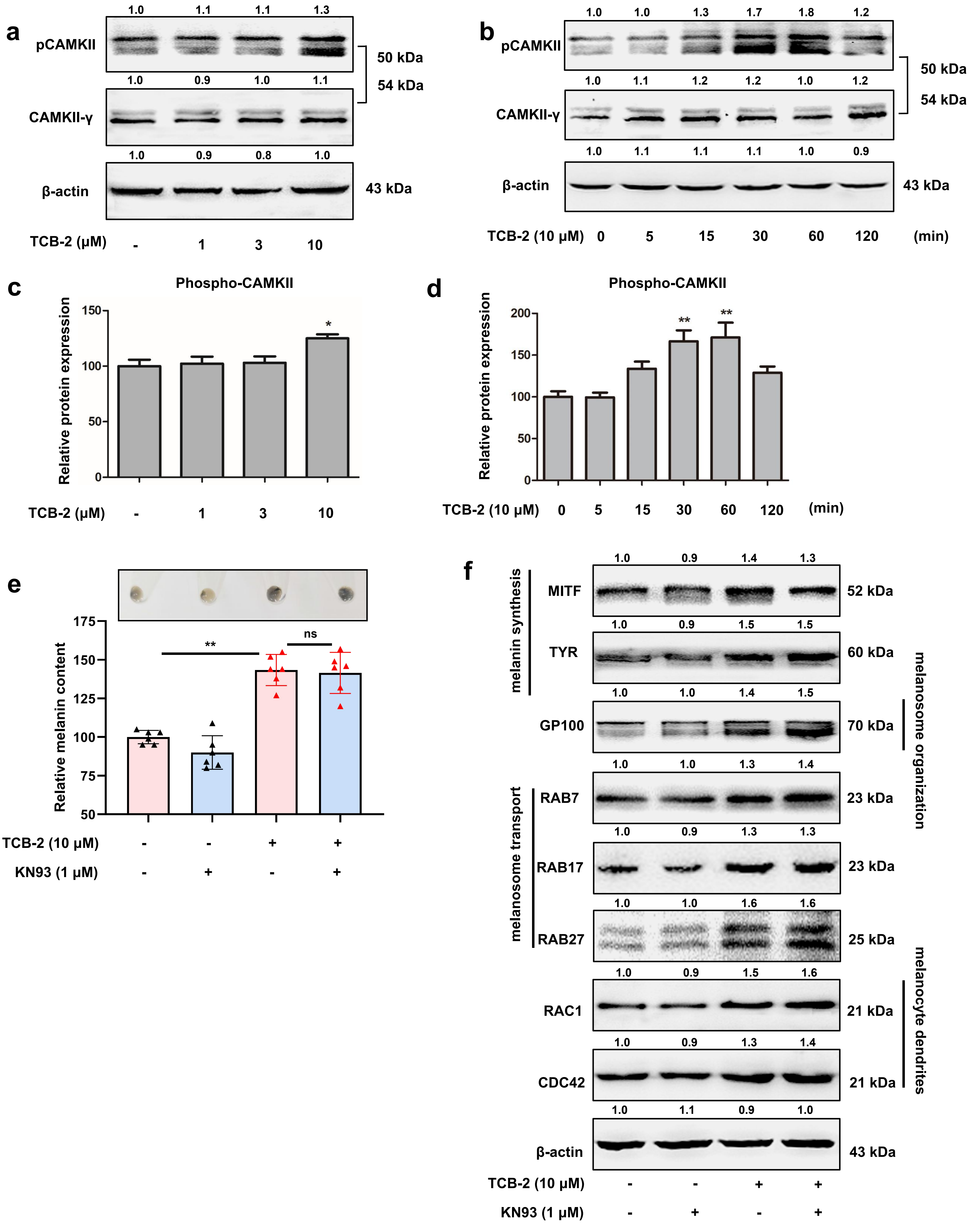
a
pCAMKII
CAMKII-γ
β-actin
1.0 1.1 1.1 1.3
1.0 0.9 1.0 1.1
1.0 0.9 0.8 1.0
50 kDa
54 kDa
43 kDa
TCB-2 (μM) - 1 3 10
b
pCAMKII
CAMKII-γ
β-actin
1.0 1.0 1.3 1.7 1.8 1.2
1.0 1.1 1.2 1.2 1.0 1.2
1.0 1.1 1.1 1.1 1.0 0.9
50 kDa
54 kDa
43 kDa
TCB-2 (10 μM) 0 5 15 30 60 120 (min)
c
Phospho-CAMKII
Relative protein expression
TCB-2 (μM) - 1 3 10
d
Phospho-CAMKII
Relative protein expression
TCB-2 (10 μM) 0 5 15 30 60 120 (min)
e
Relative melanin content
ns
TCB-2 (10 μM) - - + +
KN93 (1 μM) - + - +
f
melanin synthesis
melanosome transport
melanosome organization
melanocyte dendrites
MITF 52 kDa
TYR 60 kDa
GP100 70 kDa
RAB7 23 kDa
RAB17 23 kDa
RAB27 25 kDa
RAC1 21 kDa
CDC42 21 kDa
β-actin 43 kDa
TCB-2 (10 μM) - - + +
KN93 (1 μM) - + - +

Figure S10

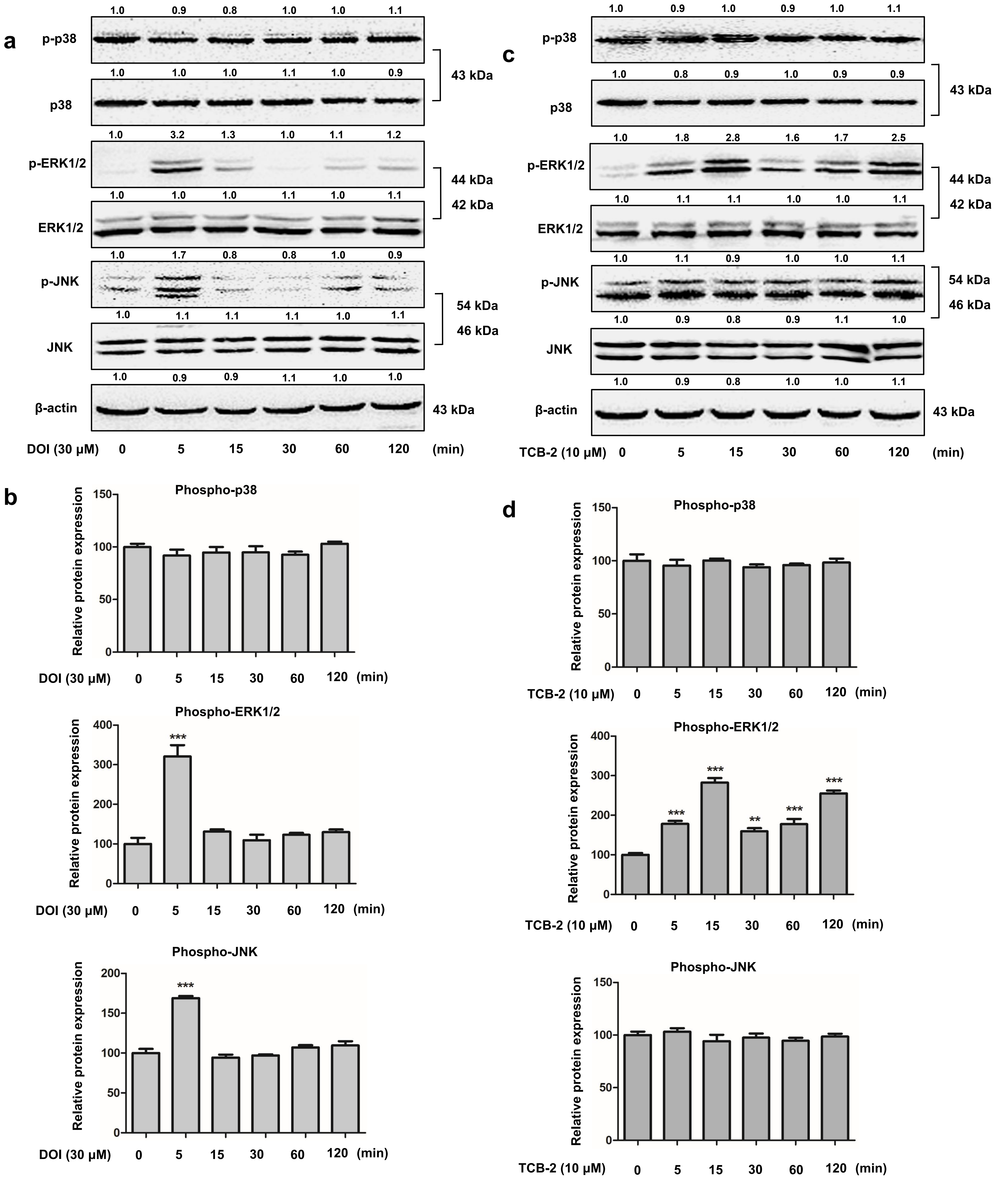
a
p-p38
1.0 0.9 0.8 1.0 1.0 1.1
p38
1.0 1.0 1.0 1.1 1.0 0.9
43 kDa
p-ERK1/2
1.0 3.2 1.3 1.0 1.1 1.2
ERK1/2
1.0 1.0 1.0 1.1 1.0 1.1
44 kDa
42 kDa
p-JNK
1.0 1.7 0.8 0.8 1.0 0.9
JNK
1.0 1.1 1.1 1.1 1.0 1.1
54 kDa
46 kDa
β-actin
1.0 0.9 0.9 1.1 1.0 1.0
43 kDa
DOI (30 μM) 0 5 15 30 60 120 (min)
c
p-p38
1.0 0.9 1.0 0.9 1.0 1.1
p38
1.0 0.8 0.9 1.0 0.9 0.9
43 kDa
p-ERK1/2
1.0 1.8 2.8 1.6 1.7 2.5
ERK1/2
1.0 1.1 1.1 1.0 1.0 1.1
44 kDa
42 kDa
p-JNK
1.0 1.1 0.9 1.0 1.0 1.1
JNK
1.0 0.9 0.8 0.9 1.1 1.0
54 kDa
46 kDa
β-actin
1.0 0.9 0.8 1.0 1.0 1.1
43 kDa
TCB-2 (10 μM) 0 5 15 30 60 120 (min)
b
Phospho-p38
Relative protein expression
DOI (30 μM) 0 5 15 30 60 120 (min)
Phospho-ERK1/2
Relative protein expression
***
DOI (30 μM) 0 5 15 30 60 120 (min)
Phospho-JNK
Relative protein expression
***
DOI (30 μM) 0 5 15 30 60 120 (min)
d
Phospho-p38
Relative protein expression
TCB-2 (10 μM) 0 5 15 30 60 120 (min)
Phospho-ERK1/2
Relative protein expression
*** *** ** *** ***
TCB-2 (10 μM) 0 5 15 30 60 120 (min)
Phospho-JNK
Relative protein expression
TCB-2 (10 μM) 0 5 15 30 60 120 (min)

Figure S11

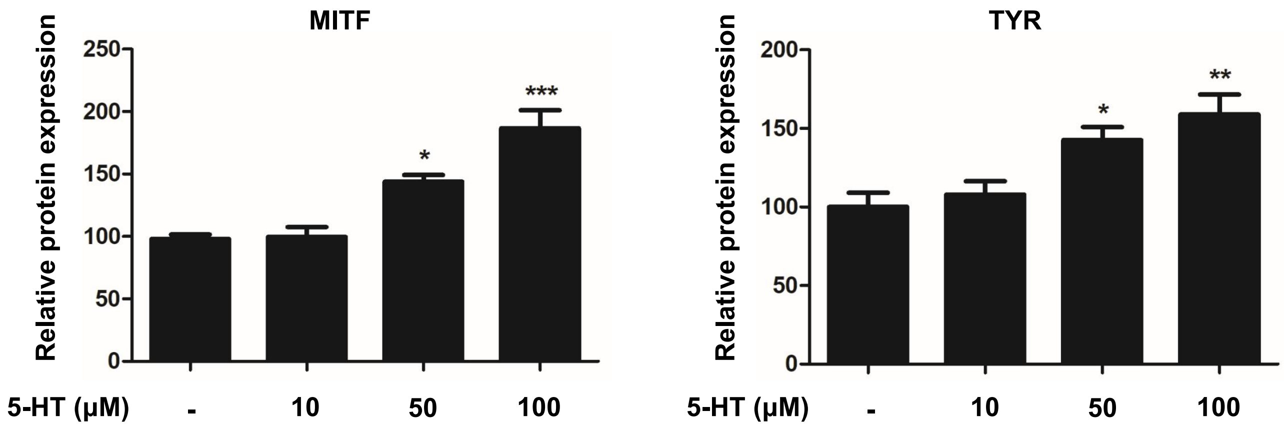

Related to Fig. 1D

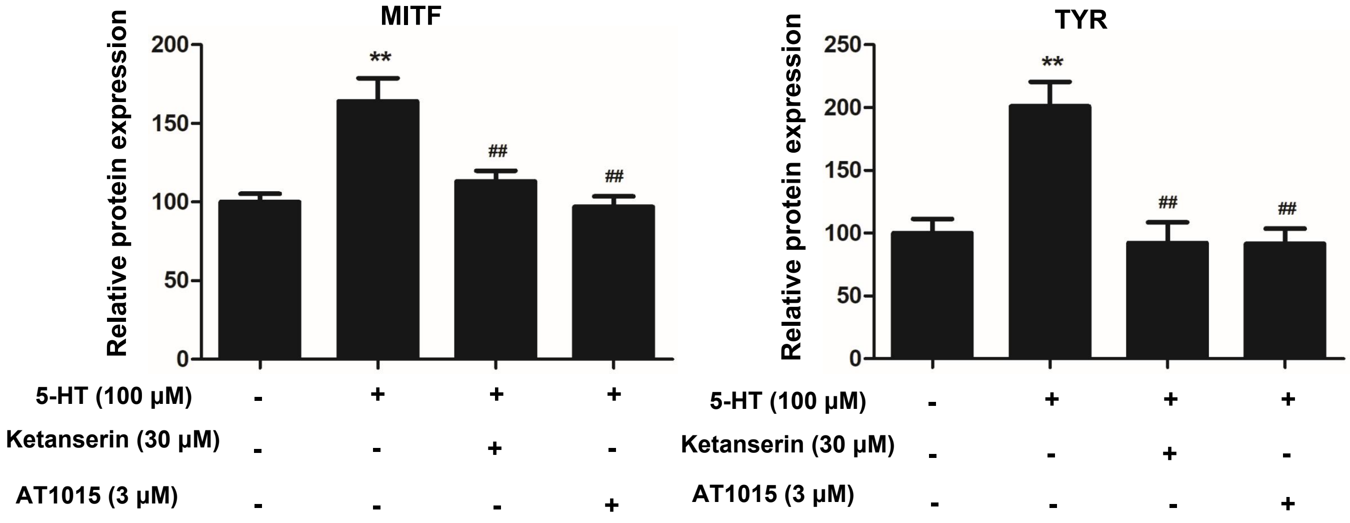

Related to Fig. S2E

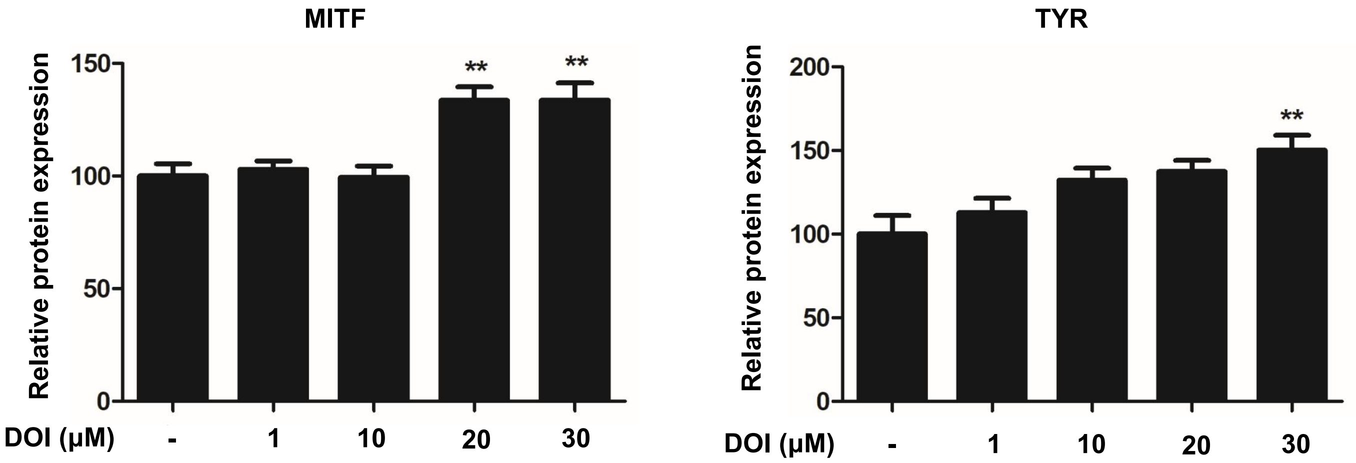

Related to Fig. 3D

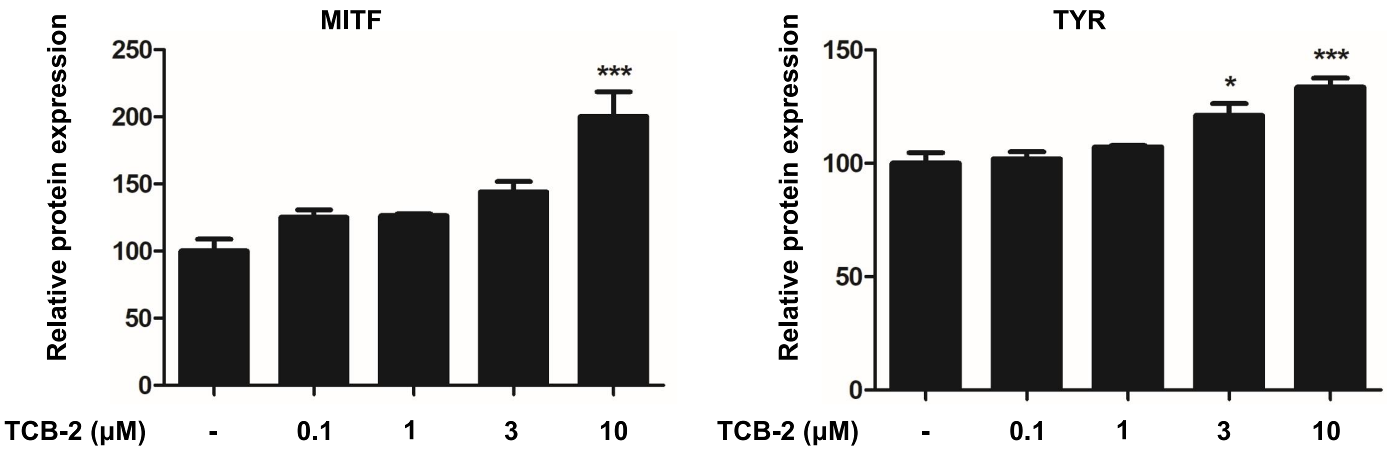

Related to Fig. S4D

Figure S12

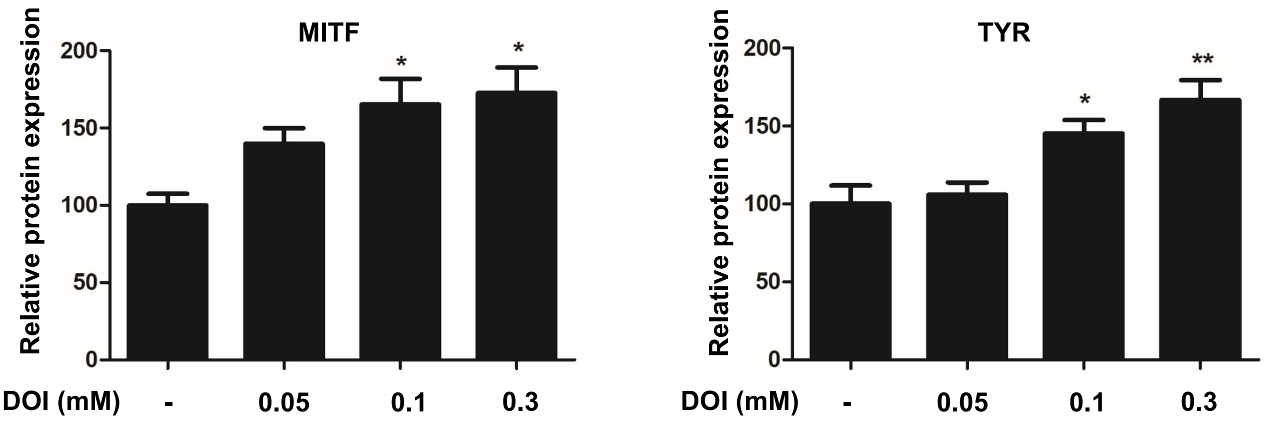

Related to Fig. 4C

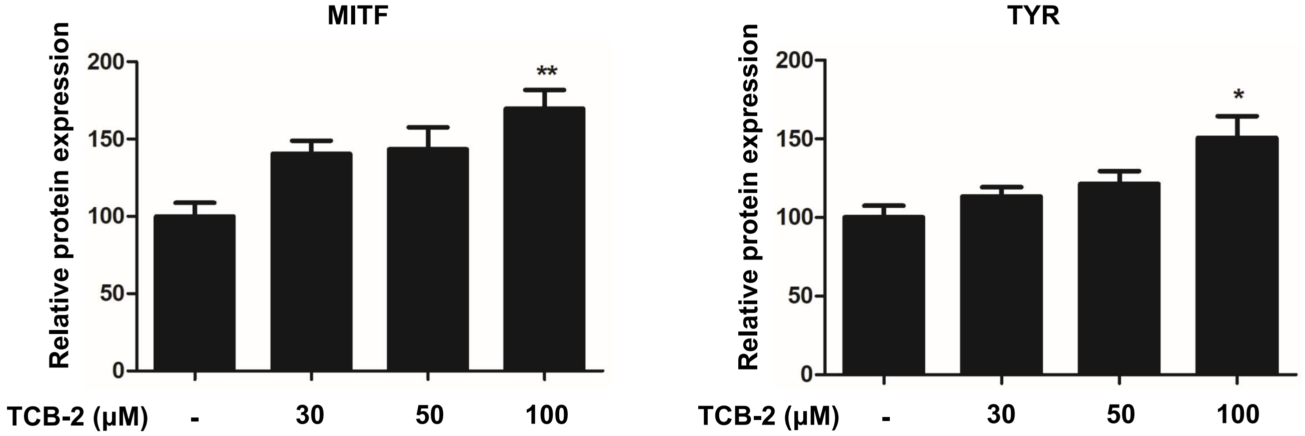

Related to Fig. S5C

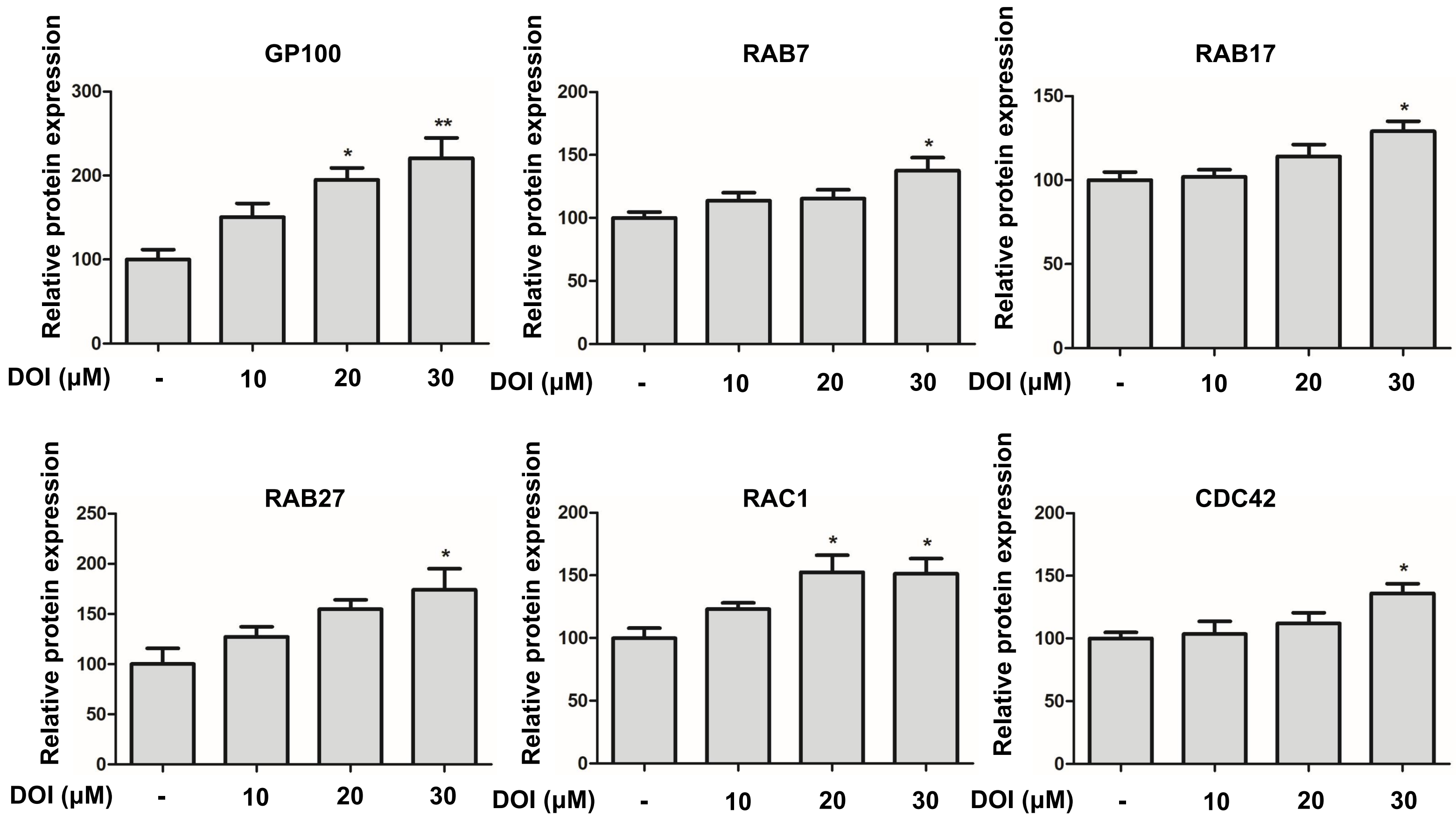

Related to Fig. 6D

Figure S13

Related to Fig. 7D

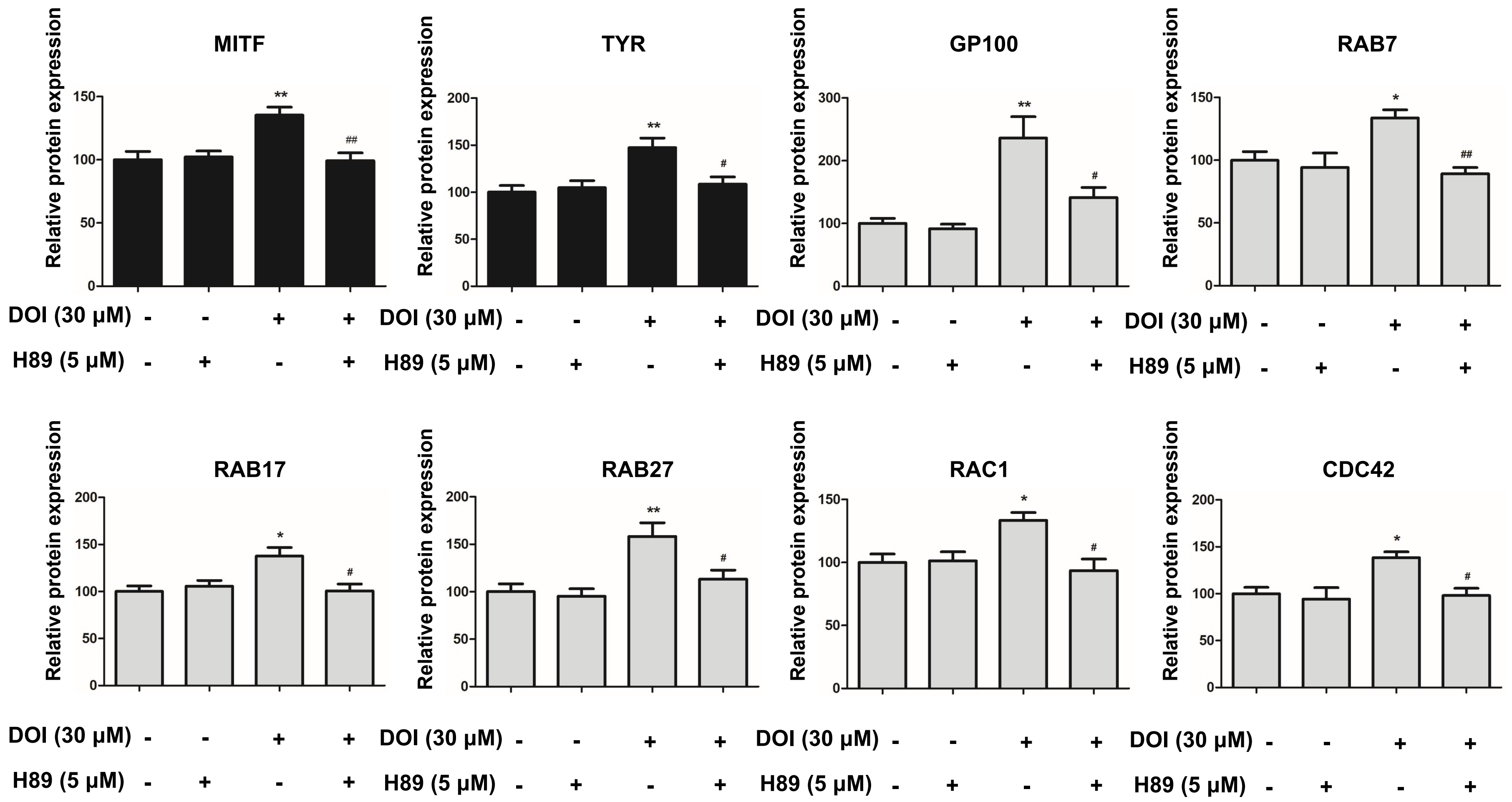

Related to Fig. S8E

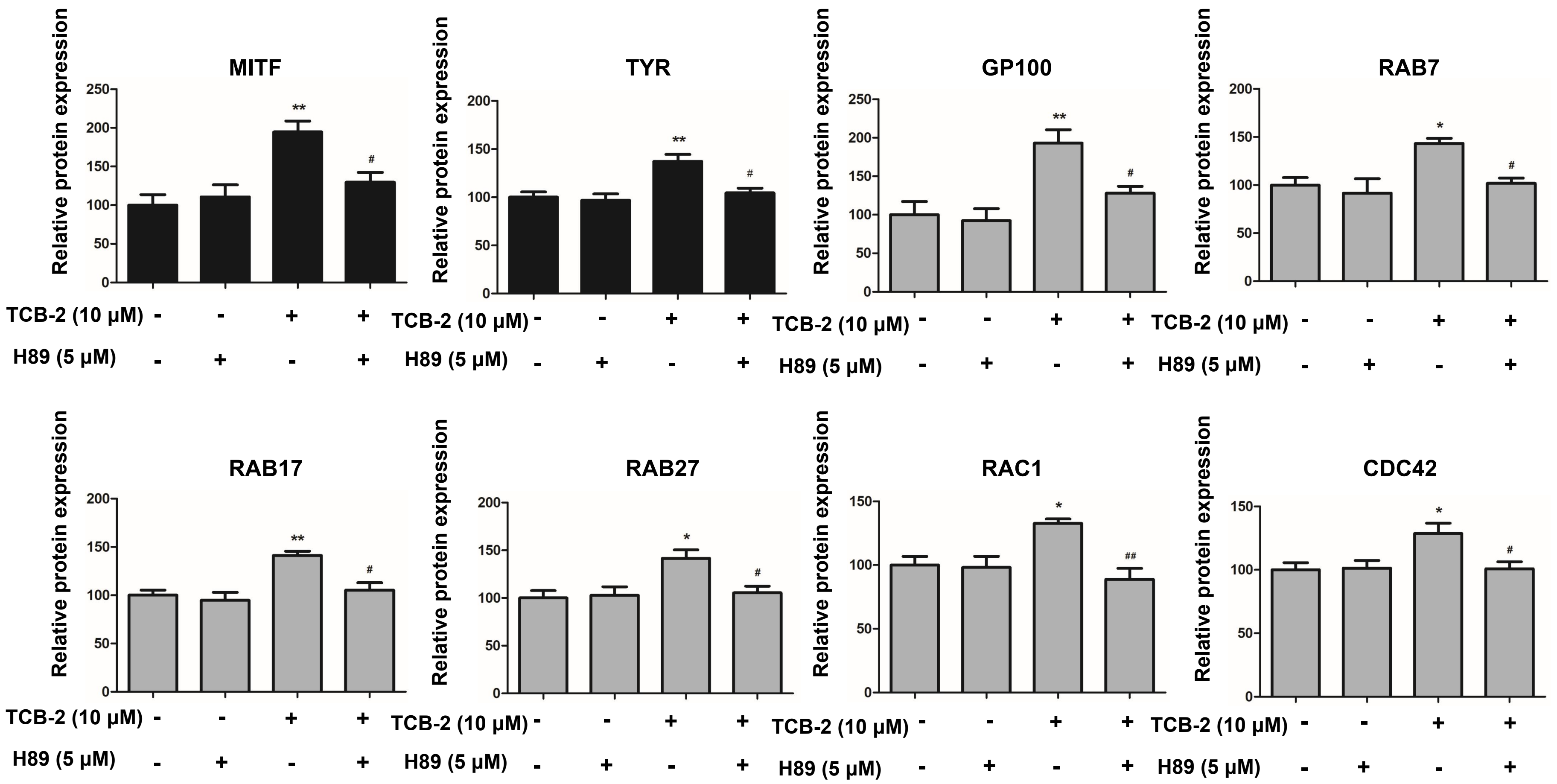

Figure S14

Related to Fig. S9F

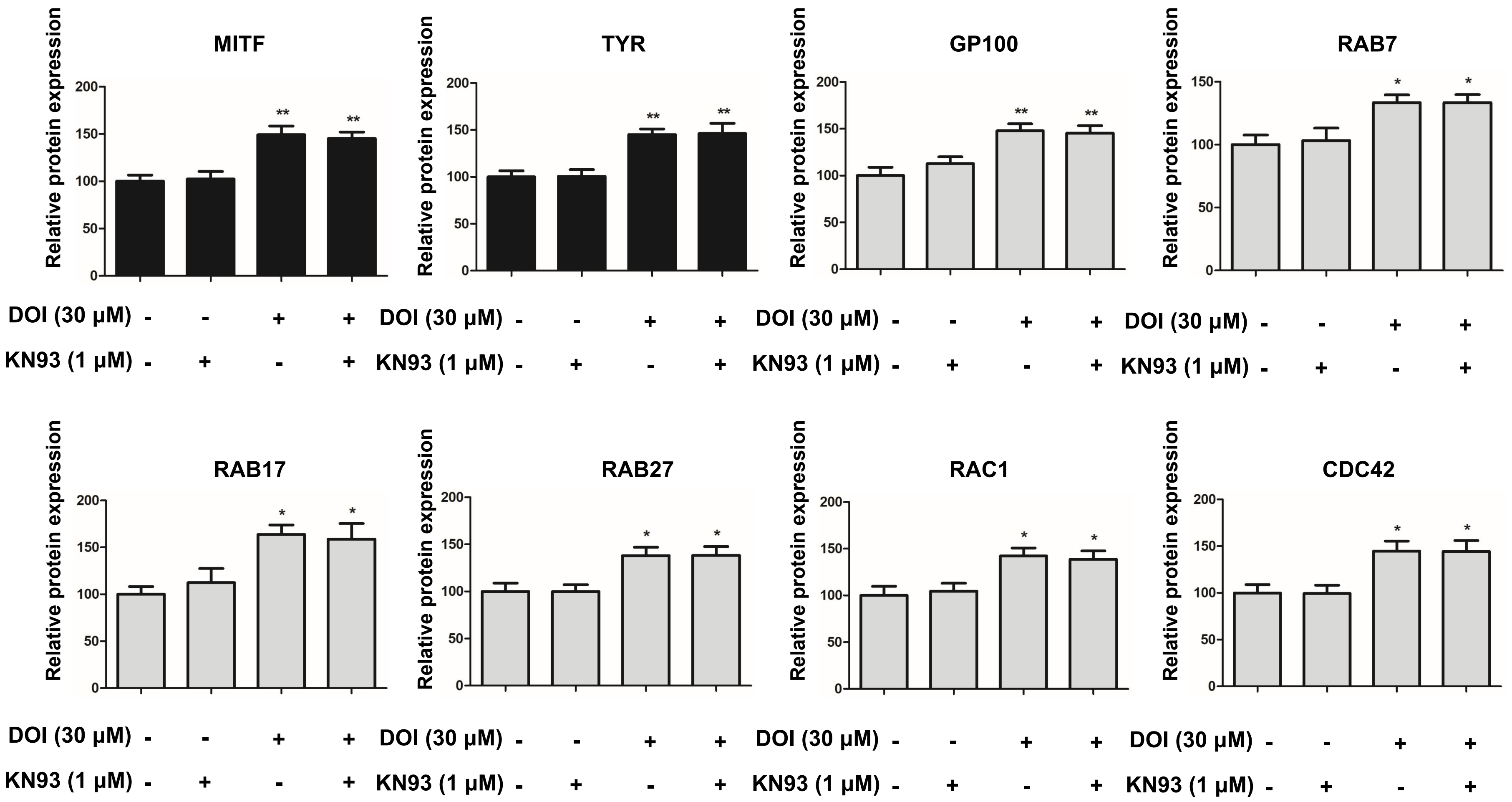

Related to Fig. S10F

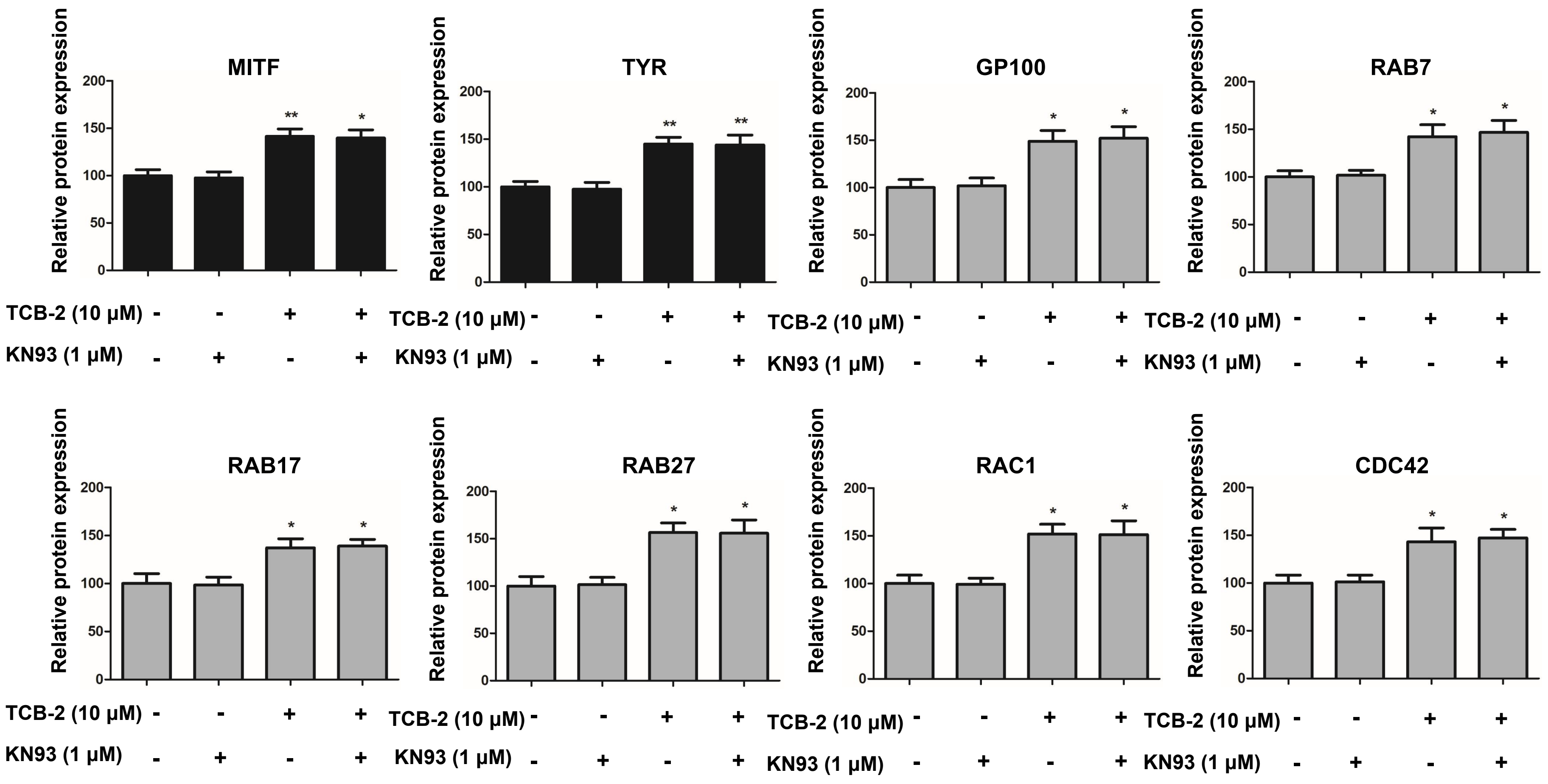

Supplement: Supplementary file 1 [file ijms-23-06111-s001.zip › ijms-1679778-SI.pdf]
